# Thioredoxin protects against diabetic hearing loss by regulating TOMM22 mediated mitochondrial autophagy in hair cells and inhibiting microglial M1 polarization

**DOI:** 10.1038/s41598-026-44909-3

**Published:** 2026-03-20

**Authors:** Shiwen Zhong, Meng Xu, Quanxiang Wang, Sifan Wang, Xiang Li, Yan Guo, Hui Kong

**Affiliations:** 1 Department of Otorhinolaryngology of the Second Hospital, Dalian Medical University, Dalian, 116023 LiaoNing China; 2 Vrtigo Clinic/Research Center of Aerospace Medicine, Air Force Medical Center, PLA, Beijing, 100142 China

**Keywords:** Diabetic hearing loss, Thioredoxin, Mitochondrial autophagy, Microglial polarization, Cell biology, Diseases, Molecular biology, Neuroscience

## Abstract

Hearing loss is a prevalent yet mechanistically unclear complication of diabetes. This research aims to systematically investigate the role of thioredoxin (Trx) in diabetic hearing loss and elucidate its underlying molecular mechanisms through clinical, bioinformatics, and experimental analyses. Clinically, elevated serum Trx levels were associated with decreased otoacoustic emission (OAE) parameters, suggesting its potential as a serum biomarker. Bioinformatics analysis revealed that TOMM22 is critical for mitophagy. In vivo, Trx overexpression effectively mitigated the hyperactivation of apoptosis, mitophagy, and M1 microglial activation in diabetic mouse cochlear tissue. An in vitro advanced glycation end-product (AGE)-induced HEI-OC1 cell model demonstrated that Trx1 overexpression inhibited PINK1/Parkin-mediated mitophagy, increased the mitochondrial membrane potential, and reduced apoptosis by maintaining TOMM22 expression. Conversely, CCCP-mediated inhibition of TOMM22 reversed the protective effects of Trx1. Moreover, Trx1 drove microglial polarization towards the anti-inflammatory M2 phenotype. Furthermore, coculture of microglia conditioned medium with HEI-OC1 cells confirmed that Trx1 indirectly protects auditory cells. Thus, Trx protects hearing via dual mechanisms: cell autonomously by regulating the TOMM22/PINK1 axis to maintain mitochondrial homeostasis and noncell autonomously by inducing microglial M2 polarization to improve the auditory microenvironment. These findings provide novel insights into diabetic hearing loss pathogenesis and identify Trx as a potential therapeutic target.

**Supplementary Information:**

The online version contains supplementary material available at 10.1038/s41598-026-44909-3.

## Introduction

 Diabetes mellitus is an endocrine metabolic disorder caused by absolute or relative insulin secretion deficiency (type 1) and/or insulin utilization disorder (type 2), which leads to a chronic increase in blood glucose levels due to carbohydrate, fat and protein metabolism disorders^1^. Prolonged and consistently elevated blood sugar levels can result in damage, functional impairments, and potentially the failure of multiple organs. The long-term complications associated with this condition, including kidney disease, microvascular disease, retinopathy, hearing loss, and peripheral neuropathy, can lead to even more severe consequences^2^. In 2021, approximately 529 million adults worldwide suffered from diabetes. It is projected that the number of patients will increase to 1.3 billion by 2050^3^. Diabetic deafness, a notable complication of diabetes, is characterized primarily by symmetrical sensorineural hearing loss in the bilateral high-frequency range and the sporadic loss of outer hair cells within the cochlea. The resulting communication barriers significantly impact the daily lives of individuals with diabetes. Through audiological examinations of elderly patients with type 2 diabetes, Frisina et al. reported that elevated blood glucose is a potential mechanism for peripheral and central hearing loss in diabetes patients^4^. In 2006, Fukushima et al. analysed temporal bone specimens from diabetic patients and reported significantly fewer outer hair cells (OHCs) in the cochlea and spiral ganglion cells (SGCs) in the inner ear^5^. In clinical practice, hearing loss in elderly diabetic patients is frequently attributed to age-related deterioration of auditory function. This misattribution can delay timely intervention and treatment during the early stages of cochlear damage. Once the condition advances to a level that impairs normal communication, it is often irreversible. Therefore, elucidating the mechanisms by which diabetes induces cochlear damage is crucial for the early diagnosis and treatment of diabetic deafness.

The mechanisms through which diabetes or a high-glucose environment leads to various complications include metabolic disorders, microvascular lesions, neurotrophic factor deficiency, and oxidative stress^6,7^. Among these, oxidative stress has garnered the most research attention regarding diabetic complications^8^. Both diabetes and a high-glucose environment can increase oxidative stress levels in the body, resulting in the excessive production of reactive oxygen species and free radicals within mitochondria. This overproduction results in oxidative damage to proteins, lipids, and nucleic acids. The resulting damage and metabolites activate the apoptotic pathway, inducing programmed cell death and ultimately leading to tissue and organ damage^9–12^. This may represent a common mechanism through which diabetes precipitates various complications. As a highly metabolic and energy-demanding auditory organ, the survival of the inner and outer hair cells, supporting cells, and spiral ganglia of the cochlea is heavily reliant on properly functioning mitochondria. Consequently, mitochondria, which are especially vulnerable to oxidative stress, are considered critical organelles involved in hearing impairment in the context of diabetes^13–15^. When mitochondria sustain damage, cells selectively eliminate them via a specialized autophagy process known as mitochondrial autophagy, which is essential for maintaining intracellular stability. The PINK1/Parkin pathway represents the most extensively researched classical pathway of mitochondrial autophagy^16,17^. Mitochondrial damage resulting from factors such as oxidative stress can lead to a reduction in mitochondrial membrane potential. In response, PTEN-induced putative kinase 1 (PINK1) accumulates on the outer mitochondrial membrane. PINK1 then phosphorylates and activates the E3 ubiquitin ligase Parkin, which recruits autophagy receptors to the outer mitochondrial membrane, facilitating the formation of autophagosomes. The subsequent fusion of autophagosomes with lysosomes results in the degradation of mitochondria^18^. Mitochondrial autophagy serves as a double-edged sword. It facilitates the recycling of damaged mitochondria, reduces the production of reactive oxygen species, and safeguards cells against both endogenous and exogenous stimuli. Conversely, excessive activation of mitochondrial autophagy may result in cellular damage. The specific role of mitochondrial autophagy in diabetic hearing loss remains controversial, and the upstream regulatory mechanism is unclear.

The connections between neurons and glial cells within ganglia are important for regulating neural development, synaptic transmission, neurometabolic balance and neuroprotective functions^19,20^. Neuroinflammatory signals are typically a response to pathogens or foreign substances; however, recent studies have shown that mitochondria or mitochondrial components—including mitochondrial DNA, mitochondrial transcription factor A (TFAM), cardiolipin, cytochrome c, formyl peptide, high mobility B protein 1 (HMGB1), and ATP—can trigger damage responses similar to those induced by pathogens^21^. Therefore, the role of mitochondrial autophagy in neuroinflammation deserves further discussion. As the resident immune cells in the central nervous system, microglia are also present in the cochlea and play a crucial role in immune regulation^22^. These cells function as resident tissue macrophages in the inner ear under physiological conditions^23–25^. Their effective role in clearing cellular debris facilitates the regeneration of damaged cochleae^26^. In the resting state, microglia can release proinflammatory factors, such as IL-6 and TNF-α, via polarization towards the M1 phenotype, while they can secrete anti-inflammatory factors, including IL-10 and IL-13, upon M2 polarization. The phenotype and function of microglia-like cells in the cochlea resemble those in the central nervous system (CNS). Excessive activation of proinflammatory microglia represents an early detrimental event that leads to cochlear cell damage^27^. In vivo and in vitro investigations of cochlear receptor cells and microglia aimed at elucidating the mechanisms underlying cochlear glial cell polarization in response to cochlear hair cell injury may establish a critical foundation for the effective prevention of hearing impairment.

Thioredoxin (Trx) is a crucial endogenous antioxidant protein that plays a central role in maintaining redox balance within cells^28^. Trx has two phenotypes: Trx1, which is expressed in the cytoplasm, and Trx2, which is expressed in the mitochondria. Under oxidative stress conditions, Trx1 can be translocated to the mitochondria to preserve their structure and function^29^. Trx not only directly scavenges reactive oxygen species but also modulates gene expression by reducing the expression of transcription factors such as NF-κB and AP-1, which are involved in various physiological and pathological processes, including cell proliferation, apoptosis, and the inflammatory response^30–32^. Recent studies indicate that Trx plays a multifaceted role in the onset and progression of diabetes and its associated complications. In individuals with diabetic retinopathy and diabetic nephropathy, dysfunction of the Trx system is closely linked to oxidative stress-related injury^33,34^. The degradation product of Trx, Trx80, has been shown to induce microglia to polarize into the M1 phenotype and exert proinflammatory effects in vivo^35^. However, the relationship between Trx and diabetic hearing impairment has yet to be investigated. Furthermore, the role of Trx in antioxidant defence and mitochondrial quality control within the cochlea remains unclear.

In this study, the correlation between serum Trx levels and audiological parameters in diabetic patients was evaluated through the analysis of clinical samples. Subsequently, core pathways and key molecules were identified using bioinformatics analysis. By employing both in vivo animal models and in vitro cell experiments, the dual mechanism through which Trx protects auditory function—through the regulation of PINK1/Parkin-mediated mitochondrial autophagy and microglial polarization—was investigated. The objective of this study is to establish a new theoretical foundation for elucidating the pathophysiological processes underlying diabetic hearing loss and to identify potential new targets for its early diagnosis and intervention.

## Materials and methods

### Patients

A total of 46 patients with type 2 diabetes mellitus who visited the Department of Endocrinology at the Second Hospital of Dalian Medical University from May to August 2022 were included in the diabetes mellitus (DM) group. Additionally, 10 healthy individuals without hearing disorders who underwent physical examinations during the same period were selected as the normal control (NC) group. All 56 subjects were screened to exclude those with organic ear diseases and those who exhibited no symptoms related to vertigo. In the diabetes group, pure tone audiometry was performed at frequencies of 0.5, 1.0, 2.0, and 4.0 kHz. An average air conduction threshold (dB HL) exceeding 25 dB in either ear was classified as abnormal hearing. The participants were categorized into two groups: 19 patients were included in the normal diabetes mellitus hearing (DMHN) group and 27 patients were included in the diabetes mellitus hearing loss (DMHL) group. For each patient, the ear with the highest average pure tone audiometry (PTA) was selected for evaluation. Data from the otoacoustic emission examinations of the 56 enrolled subjects were subsequently collected.

### Bioinformatics analysis

Mitochondrial gene data for humans and mice was obtained from the MITOCARTA3.0 database (https://www.broadinstitute.org/mitocarta/mitocarta30-inventory-mammalian-mitochondrial-proteins), and autophagy regulatory factor data were obtained from the HAMdb autophagy database (http://hamdb.scbdd.com/). The intersection of these two datasets was analysed. Additionally, genetic data from the peripheral blood of a diabetic retinopathy mouse model were obtained from the GSE221521 GEO database (https://www.ncbi.nlm.nih.gov/geo/query/acc.cgi?acc=GSE221521), and different genes between groups were assessed using GEO2R for screening. Gene Ontology (GO) and Kyoto Encyclopedia of Genes and Genomes (KEGG) analyses^36–38^ were performed using the DAVID online tool (https://david.ncifcrf.gov/tools.jsp) to identify differentially expressed genes. Furthermore, protein‒protein interaction (PPI) network analysis for these genes was conducted using the STRING online platform (https://cn.string-db.org/cgi/input?sessionId=bIjo0NOcvO4C&input_page_active_form=multiple_identifiers). The resulting data were visualized with the ggplot2 (v.3.4.2) package in R (v.4.2.2) through Hiplot Pro (https://hiplot.com.cn/).

### Animals

Male C57BL/6 wild-type (WT, *n* = 34) and transgenic mice overexpressing Trx (Tg, *n* = 19), aged 4–6 weeks and weighing 18–25 g, were utilized in this study. All animals were sourced from the Specific Pathogen-Free (SPF) Experimental Animal Center at Dalian Medical University. Upon arrival, the mice underwent a one-week acclimatization period under standard housing conditions (23 ± 2 °C, 55–60% humidity, with a 12-hour alternating light and dark cycle).

### Establishment of a type 2 diabetes mouse model

The mice were randomly assigned to four groups: wild-type control (WTNC, *n* = 18), wild-type diabetic (WTDM, *n* = 16; comprising 4 mice each observed for 4 and 8 weeks and 8 mice for 12 weeks), transgenic control (TgNC, *n* = 9), and transgenic diabetic (TgDM, *n* = 10). To induce diabetes, the mice in the diabetes groups were fed a high-fat diet (60% fat, 20% carbohydrate, and 20% protein; Xietong, XTHF60) for one month, followed by a 12-hour fast. The animals were subsequently transitioned to a standard diet (11.4% fat, 67.3% carbohydrate, and 21.3% protein; Xietong, XTC01WC-001) and received intraperitoneal injections of streptozotocin (STZ; 50 mg/kg) for five consecutive days. The control mice were maintained on a standard diet throughout the study and were injected with an equal volume of citrate buffer. Body weight and blood glucose levels were measured prior to and after the modelling process and then every two weeks thereafter. Successful diabetes modelling was defined as a blood glucose concentration exceeding 16.7 mmol/L and a HOMA-IR value greater than 2.69^39–41^. At the designated endpoints of the study, the mice were deeply anaesthetized via intraperitoneal injection of sodium pentobarbital (50 mg/kg) for final auditory function assessment and blood collection. Following these procedures, the animals were humanely euthanized by exposure to a gradually increasing concentration of carbon dioxide (CO₂) in a dedicated chamber, followed by cervical dislocation. Cochlear tissues were then rapidly harvested for subsequent analysis.

### Cell culture and transfection

HEI-OC1 cells (House Ear Institute-Organ of Corti 1 cell line; OriCell^®^, M8–0401) were cultured at 37 °C in a 5% CO_2_ atmosphere in high-glucose Dulbecco’s modified Eagle’s medium (DMEM; Gibco) supplemented with 5% foetal bovine serum (FBS; Gibco BRL) and without antibiotics. To establish stably transfected cell lines, HEI-OC1 cells were seeded in 60 mm culture dishes and transfected upon reaching 70–90% confluence with Effectene Transfection Reagent (QIAGEN, 301425) according to the manufacturer’s instructions using the pIRES2-EGFP-Trx or pIRES2-EGFP-LacZ plasmids. The culture medium was replaced one day prior to transfection. After transfection, the cells were maintained in selective medium supplemented with 150 µg/mL G418 (Sigma‒Aldrich, G9570) for two weeks to select for stably transfected populations. Multiple independent clones were isolated, and successful transfection was verified by RT‒qPCR.

Stably transfected BV2 cell lines overexpressing Trx1 (BV2-Trx1) or containing the LacZ control vector (BV2-LacZ) were established through lipid-based transfection. BV2 cells were seeded in T25 flasks and transfected upon reaching 60–80% confluence using a mixture of plasmid DNA (1 µg), Enhancer (8 µL), Buffer EC (100 µL), and Effectene Transfection Reagent (25 µL) following the manufacturer’s protocol. After 18 h of incubation, the transfection mixture was replaced with fresh complete medium. To select stably transfected clones, the cells were cultured in complete medium supplemented with 600 µg/mL G418 for approximately three weeks. Monoclonal cell colonies were subsequently picked and expanded in 24-well plates for further propagation. The successful overexpression of the target gene in the BV2-Trx1 cell line was confirmed by quantitative reverse transcription polymerase chain reaction (RT‒qPCR).

### AGEs and conditioned medium treatment

AGEs were purchased (Bioss, bs-1158P) and used at a concentration of 100 µg/mL for 48 h in high-glucose medium for all tests. BV2-LacZ/Trx-AGE-conditioned medium and high-glucose medium were added to the corresponding experimental groups at a volume ratio of 3:7.

### CCK-8 assay

After the cells were counted, 5,000 cells per well were placed in a 96-well plate. Following incubation until the cells reached 50–60% confluence, they were stimulated for 48 h with various concentrations of AGEs (0, 25, 50, 75, or 100 µg/mL) or CCCP (0, 2, 4, 6, 8 or 10 µM) prepared in culture medium. After the incubation period, the medium in each well was aspirated and replaced with 100 µL of a mixture containing 10 µL of CCK-8 reagent (APExBIO, K101828133EF5E) and 90 µL of culture medium. The plate was incubated for 2 h, after which the absorbance was measured at 450 nm using a microplate reader.

### ELISA

The concentrations of target proteins in human serum samples, mouse cochlear tissue homogenates, and BV2 cell culture supernatants were quantified according to the manufacturers’ protocols. The ELISA kits used included human Trx (Kexing, F10158-A), human Trx80 (Kexing, F10201-A), and mouse Trx80 (Kexing, KX0083-MB). The assays were performed according to the instructions of the kit manual. The absorbance was measured at 450 nm using a microplate reader, and protein concentrations were determined on the basis of the corresponding standard curves.

### Western blotting

Total protein was extracted from tissues and cells using RIPA lysis buffer (SEVEN, SW103-02) and centrifuged at 12,000 × g for 15 min at 4 °C to collect the supernatant. Protein samples were then separated by SDS‒PAGE and transferred onto polyvinylidene fluoride (PVDF) membranes (Immobilon-Psq, ISEQ00010). The membranes were blocked with 5% skim milk for 1 h at room temperature and subsequently incubated overnight at 4 °C with the following primary antibodies: anti-GAPDH (Proteintech, 10494–1-AP, 1: 5000), anti-Caspase3 (Proteintech, 25128-1-AP, 1:5000), anti-Bax (Proteintech, 60267-1-Ig, 1:5000), anti-Bcl-2 (Huabio, ET1702-53, 1:1000), anti-PINK1 (Huabio, HA723021), anti-Parkin (Huabio, ET1702-60), anti-LC3B (Huabio, ET1701-65), and anti-P62 (PTMAB, PTM-6434). Following primary antibody incubation, the membranes were incubated with the corresponding horseradish peroxidase (HRP)-conjugated secondary antibodies (goat anti-mouse IgG (Proteintech, SA00001–1, 1:5000) or goat anti-rabbit IgG (Proteintech, SA00001–2, 1:5000)). The protein bands were visualized using an ECL detection system with an exposure time of 1–2 min and imaged using Image Lab software. The band intensities were quantified using ImageJ software. Cropped blot images are displayed for clarity. Loading controls were included on the same gel and processed in parallel. Full, uncropped scans of all the blots are provided in the Supplementary Information file.

### JC-1 assay

Cells were seeded in 6-well plates at a density of 2 × 10⁵ cells per well and subjected to treatment with or without AGEs or CCCP. Following treatment, the cells were washed with phosphate-buffered saline (PBS) and subsequently incubated with a mixture consisting of 1 mL of culture medium and 1 mL of JC-1 staining solution (Solarbio, M8650) for 20 min at 37 °C. After incubation, the cells were washed twice with ice-cold JC-1 staining buffer (1×), and fresh culture medium was added. The mitochondrial membrane potential was evaluated by fluorescence microscopy, with the red/green fluorescence intensity ratio serving as an indicator of the ΔΨm. A decrease in the red/green ratio signifies mitochondrial depolarization.

### Real-time quantitative PCR

Total RNA was extracted from samples using TRIzol reagent (ABclonal, RK30129), and its concentration was quantified. Complementary DNA (cDNA) was synthesized from equal amounts of total RNA using a commercial reverse transcription kit (ABclonal, RK20433) according to the manufacturer’s instructions. Real-time quantitative PCR (RT‒qPCR) was then performed using 2× Universal SYBR Green Fast qPCR Mix (ABclonal, RK21203) in a 10 µL reaction mixture containing the cDNA template and specific primers. The thermal cycling conditions were as follows: initial denaturation at 95 °C for 3 min, followed by 45 cycles of 95 °C for 5 s and 60 °C for 30 s. Melting curve analysis was conducted at the end of each reaction to confirm amplification specificity. The relative expression of target genes was normalized to that of *GAPDH* and calculated using the 2^−ΔΔCt^ method. The sequences of all the primers used are listed in Table 1.

**Table 1:** Primer sequences used in the experiments.

Gene name	Primer sequence
*GAPDH(M)-F*	CATCACTGCCACCCAGAAGACTG
*GAPDH(M)-R*	ATGCCAGTGAGCTTCCCGTTCAG
*TOMM22(M)-F*	AGAATTTGCCTGCCCCTCAT
*TOMM22(M)-R*	CCACGGTGCTTACATGTCCT
*Trx1(M)-F*	TGCATGTGGAAGTCCGAGAG
*Trx1(M)-R*	AACTGGCCACTGTGGTAGAC
*Trx2(M)-F*	TTCGGGCACTGAGATACAGG
*Trx2(M)-R*	CCATGCATCTTGCTTCCAATTCT

### Statistical analysis

All the quantitative data are presented as the mean±standard deviation (SD). Statistical analyses were performed using GraphPad Prism software (version 8.0). The normality of the data distribution was assessed using the Shapiro‒Wilk test, and the homogeneity of variance was verified by Levene’s test. For comparisons between two groups, an unpaired two-tailed Student’s t test was applied. Categorical variables were analysed using the chi-square (χ²) test. For comparisons among more than two groups, one-way analysis of variance (ANOVA) was conducted, followed by Tukey’s post hoc test for multiple comparisons. Significance levels are denoted in the figures as */#*P* < 0.05, **/##*P* < 0.01, ***/###*P* < 0.001, and ****/####*P* < 0.0001, where asterisks (*) and hash symbols (#) indicate specific intergroup comparisons as defined in the figure legends.

### Ethical approval and patient consent to participate

Human serum samples were collected as residual samples from inpatients and outpatients during their routine clinical care (such as blood tests for diagnosis or health examinations). No additional blood was drawn from any participant for the purpose of this research. The amount of serum used was part of the leftover sample from standard clinical testing and did not affect the patients’ normal diagnosis or treatment. Audiological data, including transient evoked otoacoustic emission (TEOAE) and distortion product otoacoustic emission (DPOAE), were collected as part of a standardized clinical assessment protocol for the same cohort of participants. These tests were conducted by trained audiologists using calibrated clinical equipment following established clinical guidelines. All methods were carried out in accordance with the relevant guidelines and regulations, including the principles of the Declaration of Helsinki.

All participants provided written informed consent for their deidentified residual serum samples and associated clinical data to be used for future biomedical research. The entire study protocol, encompassing sample collection, data acquisition, and usage, was reviewed and approved by the Ethics Committee of The Second Hospital of Dalian Medical University (Ethics Approval No. 2024002).

### ARRIVE guidelines for the animal experiments

This study was conducted and is reported in accordance with the Animal Research: Reporting of In Vivo Experiments (ARRIVE) guidelines 2.0. All experimental procedures received approval from the Animal Ethics and Use Committee of the SPF Animal Center at Dalian Medical University and were conducted in compliance with the relevant institutional and national guidelines for the care and use of laboratory animals. The experimental protocol received approval from the Institutional Animal Ethics and Usage Committee at the SPF Animal Centre of Dalian Medical University (Ethical code: AEE22090).

## Results

### Trx and diabetic hearing loss: innovative biomarkers

**Fig. 1:**
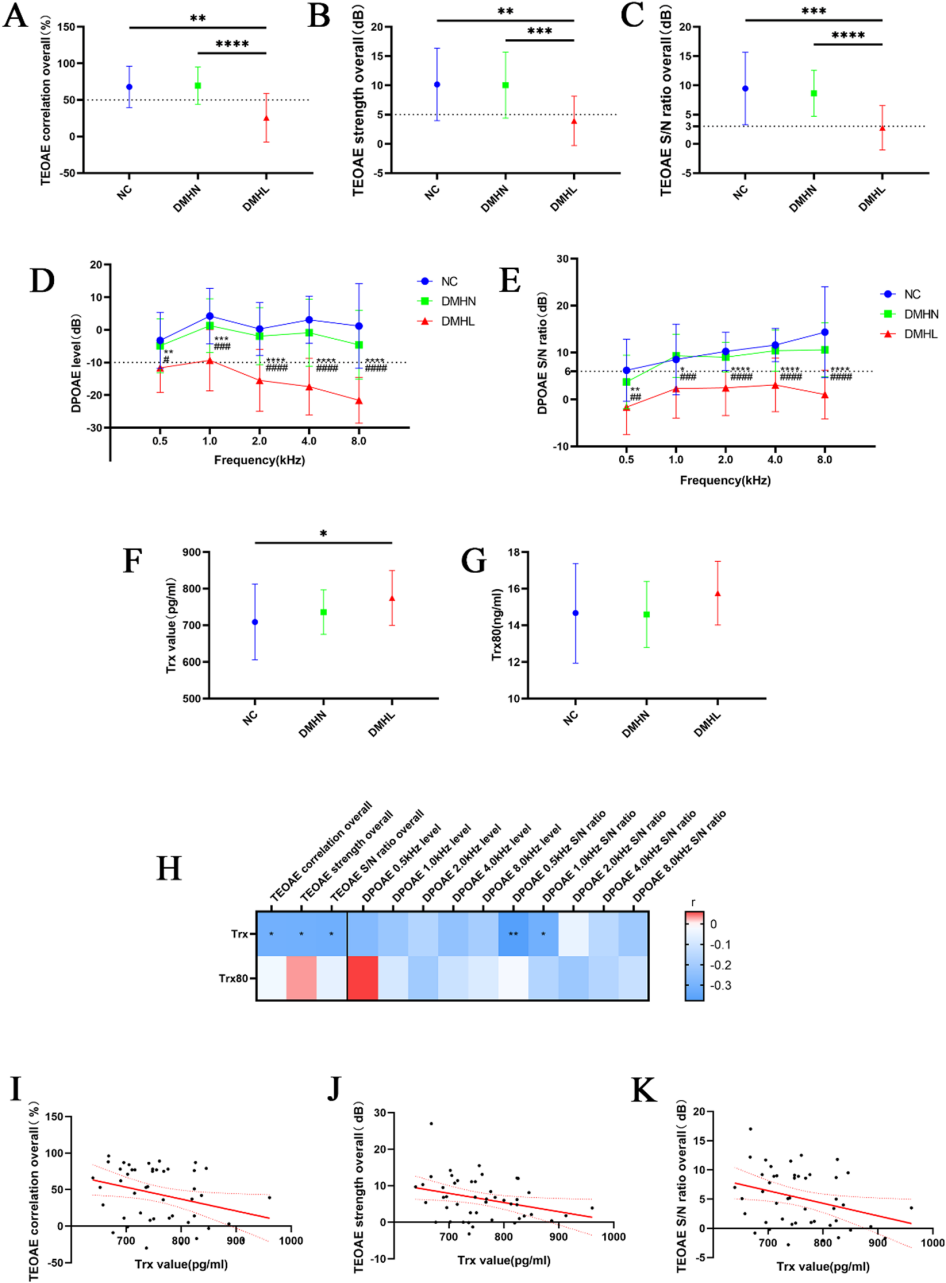
Trx and diabetic hearing loss: innovative biomarkers. (**A**–**C**) Differences in TEOAE among groups in terms of correlation, strength, and S/N ratio overall. **P < 0.01, ****P < 0.0001. (**D**,**E**) Differences in DPOAE among groups in terms of each frequency level and S/N ratio. */#*P* < 0.05, **/##*P* < 0.01, ***/###*P* < 0.001, ****/####*P* < 0.0001. (**F**,**G**) Differences in serum Trx and Trx80 levels between groups determined by ELISA. *P < 0.05. (**H**–**K**) Correlation analysis of serum Trx and Trx80 levels with TEOAE, strength, and the S/N ratio overall and correlation analysis of DPOAE at each frequency. r indicates the Pearson correlation coefficient. *P < 0.05, **P < 0.01.

Overall, the correlation, strength, and signal-to-noise ratio of the TEOAE in the DMHL group were significantly lower than those in the NC group and the DMHN group (Fig. 1A–C). The DPOAE level and signal-to-noise ratio at each frequency in the DMHL group were significantly lower than those in the NC group and the DMHN group (Fig. 1D, E). The serum Trx level in the DMHL group was significantly greater than that in the NC group but was not significantly different from that in the DMHN group. There was no significant difference in serum Trx80 levels among these groups (Fig. 1F, G). The serum Trx level in the DM group was significantly negatively correlated with the correlation, strength, and signal-to-noise ratio of the overall TEOAE and negatively correlated with the signal-to-noise ratios of DPOAE at 0.5 kHz and 1.0 kHz (Fig. 1H). These findings suggest that Trx and TEOAE are highly sensitive indicators of diabetic hearing loss. The serum Trx concentration in the DM group was significantly negatively correlated with TEOAE in terms of correlation strength and signal-to-noise ratio overall (Fig. 1I–K).

### Bioinformatics analysis of the new anchor points through which Trx regulates mitochondrial autophagy in mice with diabetic retinopathy

**Fig. 2:**
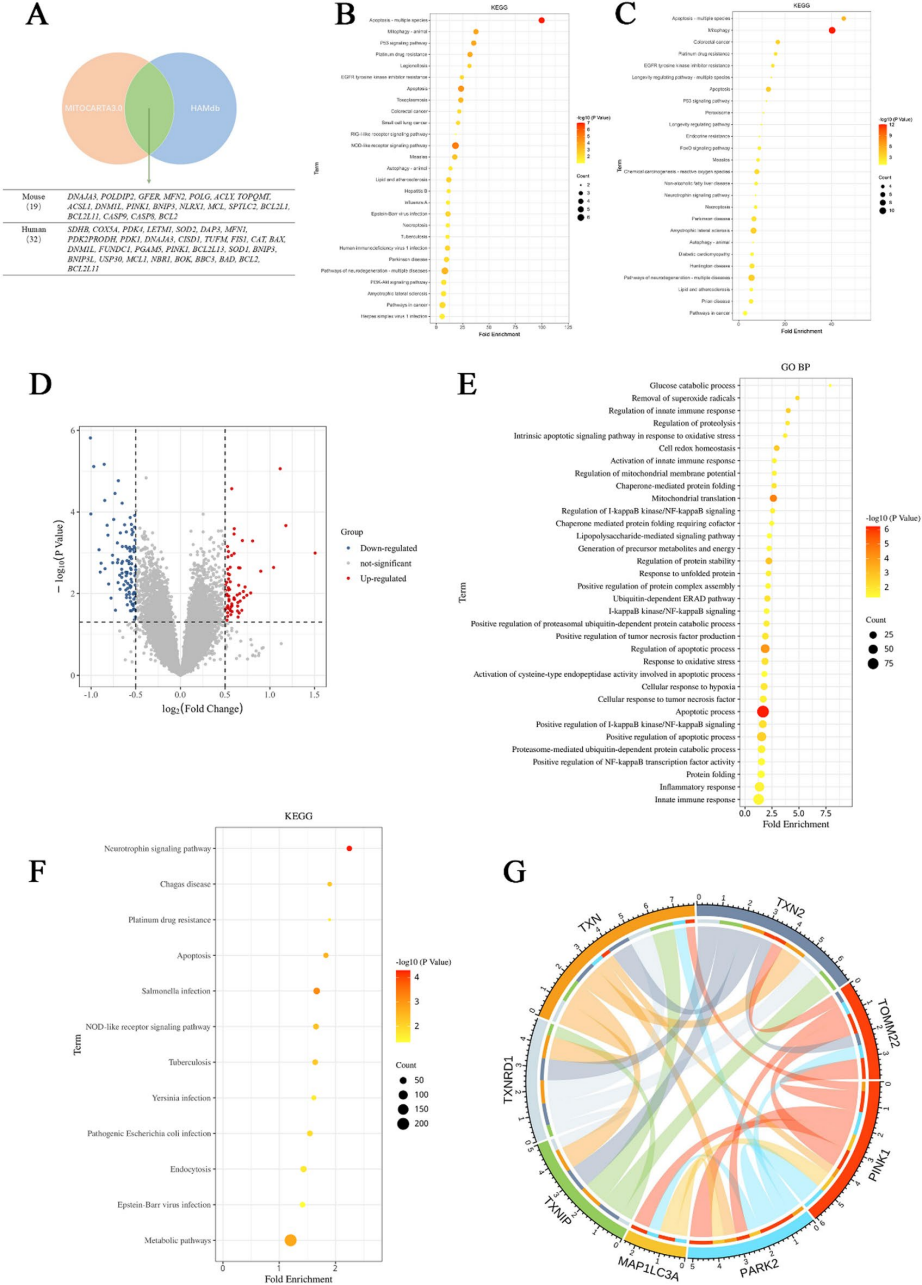
Bioinformatics analysis of the new anchor points through which Trx regulates mitochondrial autophagy in mice with diabetic retinopathy. (**A**) Intersection analysis of the data obtained from the mitochondrial database and the autophagy regulatory factor database. (**B**) KEGG analysis of human mitochondria and autophagy intersecting genes. (**C**) KEGG analysis of human mitochondria and autophagy intersecting genes. (**D**) Differentially expressed genes between the mouse model of diabetic retinopathy in GSE221521 and control group mice. (**E**) GO BP analysis of differentially expressed genes. (**F**) KEGG analysis of the differentially expressed genes. (**G**) PPI interaction network. The results of the KEGG pathway analysis are reproduced with permission from Kanehisa Laboratories. © Kanehisa Laboratories.

The mitochondrial gene data for humans and mice were obtained from the mitochondrial database MITOCARTA3.0, while the autophagy regulatory factor data for both species were sourced from the autophagy database HAMdb. Comparative analysis revealed 32 intersecting genes associated with both mitochondria and autophagy in humans, as follows: *SDHB*,* COX5A*,* PDK4*,* LETM1*,* SOD2*,* DAP3*,* MFN1*,* PDK2PRODH*,* PDK1*,* DNAJA3*,* CISD1*,* TUFM*,* FIS1*,* CAT*,* BAX*,* DNM1L*,* FUNDC1*,* PGAM5*,* PINK1*,* BCL2L13*,* SOD1*,* BNIP3*,* BNIP3L*,* USP30*,* MCL1*,* NBR1*,* BOK*,* BBC3*,* BAD*,* BCL2*,* and BCL2L11.* In mice, 19 intersecting genes related to mitochondria and autophagy were identified: *DNAJA3*,* POLDIP2*,* GFER*,* MFN2*,* POLG*,* ACLY*,* TOPQMT*,* ACSL1*,* DNM1L*,* PINK1*,* BNIP3*,* NLRX1*,* MCL*,* SPTLC2*,* BCL2L1*,* BCL2L11*,* CASP9*,* CASP8*,* and BCL2* (Fig. 2A). The intersecting genes were further analysed using KEGG to identify those involved in mitochondrial autophagy. This analysis revealed 10 genes related to human mitochondrial autophagy, namely, *MFN1*,* FIS1*,* FUNDC1*,* PGAM5*,* PINK1*,* BCL2L13*,* BNIP3*,* BNIP3L*,* USP30*, and *NBR1* (Fig. 2B), as well as four mouse mitochondrial autophagy-related genes, namely, *MFN2*,* PINK1*,* BNIP3*,* and BCL2L1* (Fig. 2C). These results indicate that the classical pathway induced by PINK1 is a shared mitochondrial autophagy pathway in both humans and mice.

Data from the mouse model dataset of diabetic retinopathy, GSE221521, were categorized into diabetes and control groups for differential analysis, resulting in the identification of 17,157 differentially expressed genes (Fig. 2D). Subsequent analyses included biological process (GO BP, Fig. 2E), cellular component (GO CC), molecular function (GO MF), and KEGG pathway analyses (Fig. 2F) to identify genes associated with mitochondria or involved in antioxidant stress responses. A protein‒protein interaction analysis was performed between the enriched differentially expressed genes from diabetic mice and the genes associated with the PINK1-mediated mitophagy pathway and Trx. The results revealed that *TOMM22* plays a crucial role in anchoring the Trx-regulated PINK1/Parkin/LC3B axis, which induces mitophagy (Fig. 2G). In the GSE221521 dataset, the expression of *TOMM22* in the peripheral blood of the diabetes group was lower than that in the control group, while *PINK1* expression increased and *LC3A* expression decreased. Additionally, the expression of *TXNIP*, TR1, and *TXN* in the peripheral blood of the diabetes group was elevated, whereas the expression of *TXN2* was reduced (Table 2).

**Table 2:** Comparison of the gene and protein names and their expression in the diabetes group.

Gene symbol	Description	Log_2_ (fold change)
*PINK1*	PTEN-induced kinase 1 (PINK1)	0.0891912
*PARK2*	PRKN E3 ubiquitin protein ligase (Parkin)	/
*MAP1LC3A*	Microtubule associated protein 1 light chain 3 alpha (LC3A)	− 0.1217147
*TXNIP*	Thioredoxin interacting protein (Txnip)	0.2386202 **
*TXNRD1*	Thioredoxin reductase 1 (TrxR1)	0.211142 *
*TXN*	Thioredoxin (Trx)	0.1582278
*TXN2*	Thioredoxin 2 (Trx2)	− 0.1695524 *
*TOMM22*	Translocase of outer mitochondrial membrane 22 (TOMM22)	− 0.1486933 *

A log_2_(fold change) > 0 indicates increased expression of the differentially expressed gene in the diabetes group, and a log_2_(fold change) < 0 indicates decreased expression of the differentially expressed gene in the diabetes group, indicating that this gene was considered to be differentially expressed; *P < 0.05, **P < 0.01 ***P < 0.001, and ****P < 0.0001.

### The protective effect of Trx overexpression on cochlear apoptosis and mitochondrial autophagy in diabetic mice

**Fig. 3:**
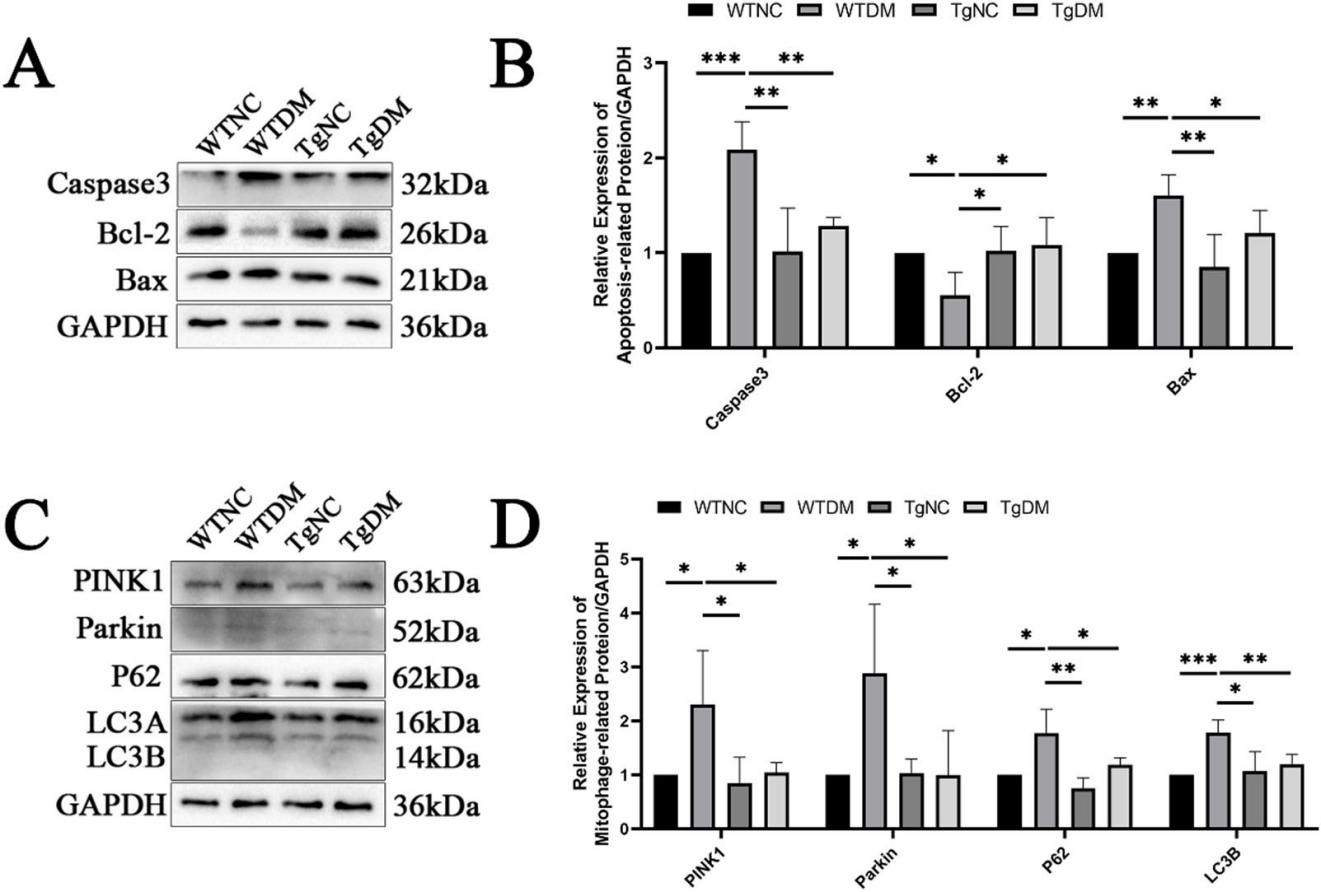
Pprotective effect of Trx overexpression on cochlear apoptosis and mitochondrial autophagy in diabetic mice. (**A**,**B**) The expression of the pro-apoptotic proteins Caspase3 and Bax and the expression of the anti-apoptotic protein Bcl-2 in the cochlear tissues of mice determined by Western blotting. (**C**,**D**) The expression of the mitochondrial autophagy pathway proteins PINK1, Parkin, P62 and LC3 in the cochlear tissues of mice was determined by Western blotting. Western blotting and image presentation: Cropped blot images are displayed for clarity and conciseness. Within each horizontal panel, the lanes are contiguous on the original blot, representing samples with proteins of similar molecular weight analysed on the same gel. Vertically arranged panels group cropped sections from the same original membrane that were probed for different target proteins with distinct molecular weights. These vertical groups are explicitly separated by black horizontal dividing lines. Full, uncropped scans of all original blots, with groups clearly labelled, are provided in the Supplementary Information. *P < 0.05, **P < 0.01, ***P < 0.001.

The expression levels of the pro-apoptotic proteins Caspase3 and Bax, as well as the anti-apoptotic protein Bcl-2, were assessed in the cochlear tissues of mice using Western blotting. Compared with those in the WTNC group, the expression levels of Caspase3 and Bax were elevated in the WTDM group, whereas the expression of Bcl-2 was reduced. Conversely, when the WTDM group was compared to the TgDM group, the levels of Caspase3 and Bax decreased, whereas the expression of Bcl-2 increased (Fig. 3A,B). Additionally, the expression of the mitochondrial autophagy pathway proteins PINK1, Parkin, P62, and LC3B was evaluated in cochlear tissues. Compared with those in the WTNC group, the expression levels of PINK1, Parkin, P62, and LC3B were increased in the WTDM group. In contrast, compared with the WTDM group, the TgDM group exhibited decreased expression of PINK1, Parkin, P62, and LC3B (Fig. 3C, D).

### AGEs induced apoptosis and mitochondrial autophagy in HEI-OC1 cells

**Fig. 4:**
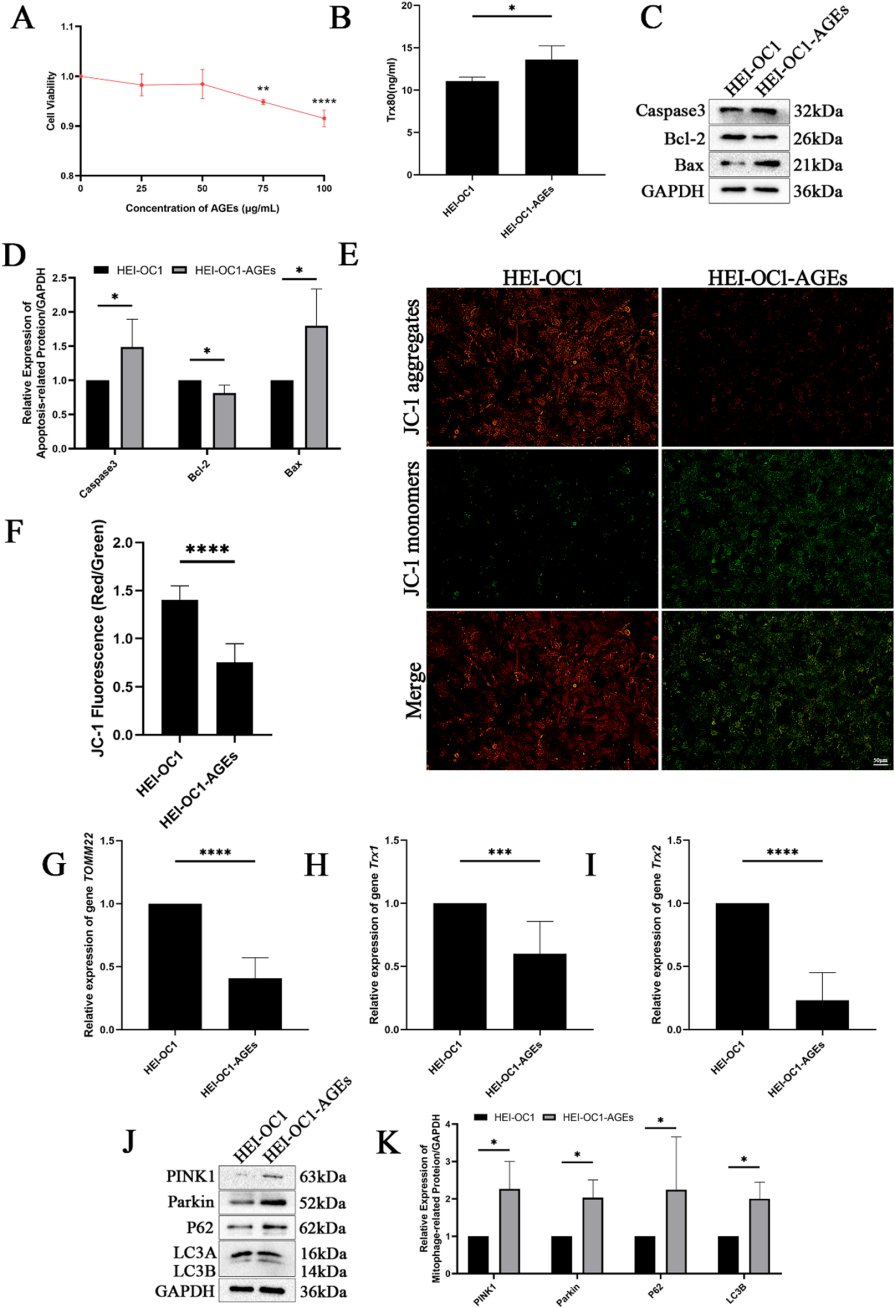
AGEs induced apoptosis and mitochondrial autophagy in HEI-OC1 cells. (**A**) The optimal concentration of AGEs for 48 h of incubation was determined by CCK-8 assays. (**B**) Expression of Trx80 in HEI-OC1 cells treated with AGEs was determined by ELISA. (**C**,**D**) Expression of apoptotic proteins in HEI-OC1 cells induced by AGEs was determined by Western blotting. (**E**,**F**) Mitochondrial membrane potential in HEI-OC1 cells treated with AGEs was determined by a JC-1 assay (Scale bar=50 μm. Images were taken at ×20 magnification). (**G**) Expression of *TOMM22* in HEI-OC1 cells treated with AGEs was determined by RT‒qPCR. (**H**) Expression of *Trx1* in HEI-OC1 cells treated with AGEs was determined by RT‒qPCR. (**I**) Expression of *Trx2* in HEI-OC1 cells treated with AGEs was determined by RT‒qPCR. (**J**,**K**) Expression of mitochondrial autophagy-related proteins in AGE-induced HEI-OC1 cells was determined by Western blotting. Western blotting and image presentation: Cropped blot images are displayed for clarity and conciseness. Within each horizontal panel, the lanes are contiguous on the original blot, representing samples with proteins of similar molecular weight analysed on the same gel. Vertically arranged panels group cropped sections from the same original membrane that were probed for different target proteins with distinct molecular weights. These vertical groups are explicitly separated by black horizontal dividing lines. Full, uncropped scans of all original blots, with groups clearly labelled, are provided in the Supplementary Information. *P < 0.05, **P < 0.01, ***P < 0.001, **** *P* < 0.0001.

Oxidative stress damage was induced in HEI-OC1 cells by AGEs. To identify the optimal concentration of AGEs for subsequent experiments, we treated HEI-OC1 cells with a gradient of concentrations (0, 25, 50, 75, and 100 µg/mL) for 48 h and assessed cell viability using a CCK-8 assay. As shown in Fig. 4A, AGEs inhibited cell proliferation in a dose-dependent manner. While a significant reduction in viability was observed at 75 µg/mL, the maximum inhibitory effect was achieved at 100 µg/mL. Consequently, 100 µg/mL was selected as the optimal concentration for all subsequent functional assays to ensure a robust and reproducible biological response. The ELISA results indicated that the level of Trx80 in HEI-OC1 cells increased in response to AGEs (Fig. 4B). Western blotting revealed that AGEs increased the expression of the pro-apoptotic proteins Caspase3 and Bax in HEI-OC1 cells, whereas the expression of the anti-apoptotic protein Bcl-2 decreased (Fig. 4C, D). Changes in mitochondrial membrane potential were assessed using a JC-1 assay. The findings of this assay demonstrated that AGEs led to a reduction in the red/green fluorescence ratio in HEI-OC1 cells, suggesting a decrease in the mitochondrial membrane potential (Fig. 4E, F). RT‒qPCR revealed that the expression of *TOMM22*,* Trx1* and *Trx2* in HEI-OC1 cells decreased upon exposure to AGEs (Fig. 4G–I). The expression of mitochondrial autophagy-related proteins was subsequently evaluated through Western blotting, and the results indicated that AGEs increased the expression of the mitochondrial autophagy pathway proteins PINK1, Parkin, P62, and LC3B in HEI-OC1 cells (Fig. 4J, K).

### Protective effect of Trx1 overexpression on apoptosis and mitochondrial autophagy in HEI-OC1 cells treated with AGEs

**Fig. 5:**
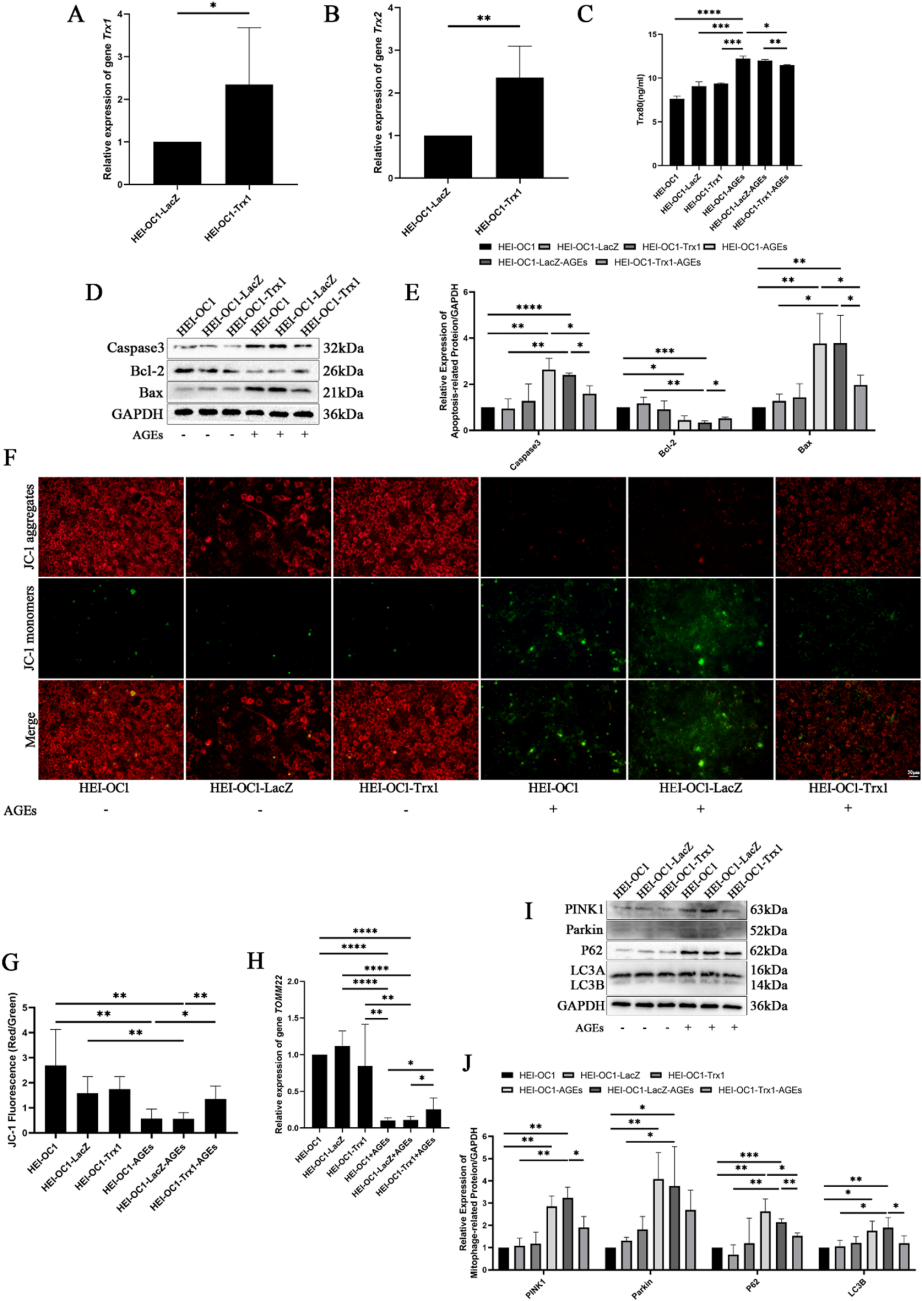
Pprotective effect of Trx1 overexpression on apoptosis and mitochondrial autophagy in HEI-OC1 cells treated with AGEs. (**A**) *Trx1* expression in HEI-OC1-Trx1 cells was determined by RT‒qPCR. (**B**) *Trx2* expression in HEI-OC1-Trx1 cells was determined by RT‒qPCR. (**C**) Trx80 expression in HEI-OC1 cells regulated by Trx1 and treated with AGEs was determined by ELISA. (**D**,**E**) Expression of apoptotic proteins in HEI-OC1 cells regulated by Trx1 treated with AGEs was determined by Western blotting. (**F**,**G**) Changes in the mitochondrial membrane potential of HEI-OC1 cells regulated by Trx1 treated with AGEs was determined by a JC‒1 assay (Scale bar=50 μm. Images were taken at ×20 magnification). (**H**) *TOMM22* expression in HEI-OC1 cells regulated by Trx1 treated with AGEs was determined by RT‒qPCR. (**I**,**J**) Expression of mitochondrial autophagy-related proteins in HEI-OC1 cells regulated by Trx1 treated with AGEs was determined by Western blotting. Western blotting and image presentation: Cropped blot images are displayed for clarity and conciseness. Within each horizontal panel, the lanes are contiguous on the original blot, representing samples with proteins of similar molecular weight analysed on the same gel. Vertically arranged panels group cropped sections from the same original membrane that were probed for different target proteins with distinct molecular weights. These vertical groups are explicitly separated by black horizontal dividing lines. Full, uncropped scans of all original blots, with groups clearly labelled, are provided in the Supplementary Information. *P < 0.05, **P < 0.01, ***P < 0.001, **** *P* < 0.0001.

The expression levels of Trx1 and Trx2 in HEI-OC1 cells transfected with the Trx1 gene were assessed using RT‒qPCR. The results indicated that the expression levels of *Trx1* and *Trx2* in HEI-OC1-Trx1 cells were significantly increased compared with those in HEI-OC1-LacZ cells (Fig. 5A, B). ELISA revealed a significant increase in the Trx80 content in both HEI-OC1-AGEs and Hei-OC1-LacZ-AGEs following treatment with AGEs. In contrast, the Trx80 content in HEI-OC1-Trx1-AGEs was lower than that in HEI-OC1-AGEs and HEI-OC1-LacZ-AGEs (Fig. 5C). The expression of apoptosis-related proteins was analysed via Western blotting. Compared with those in the HEI-OC1 group, the levels of the proapoptotic proteins Caspase3 and Bax in the HEI-OC1-AGEs and HEI-OC1-LacZ-AGEs groups were greater, whereas the expression of the antiapoptotic protein Bcl-2 was lower. In HEI-OC1-Trx1-AGEs, the expression of Caspase3 and Bax was lower than that in HEI-OC1-AGEs and HEI-OC1-LacZ-AGEs, whereas the expression of Bcl-2 was greater than that in HEI-OC1-LacZ-AGEs (Fig. 5D, E). A JC-1 assay was used to assess alterations in mitochondrial membrane potential. The results indicated a decrease in the red/green fluorescence ratio of HEI-OC1-AGEs compared with that in HEI-OC1 cells. Additionally, the red/green fluorescence ratio of HEI-OC1-LacZ-AGEs was lower than that in both HEI-OC1 and HEI-OC1-LacZ cells. Notably, the red/green fluorescence ratio in HEI-OC1-Trx1-AGEs was greater than that in HEI-OC1-AGEs and HEI-OC1-LacZ-AGEs (Fig. 5F, G). RT‒qPCR revealed that the expression of *TOMM22* in HEI-OC1-AGEs and HEI-OC1-LacZ-AGEs was lower than that in HEI-OC1 and HEI-OC1-LacZ cells. Furthermore, *TOMM22* expression in HEI-OC1-Trx1-AGEs was greater than that in HEI-OC1-AGEs and HEI-OC1-LacZ-AGEs (Fig. 5H). The expression of mitochondrial autophagy proteins was subsequently evaluated via Western blotting. The findings demonstrated that, compared with those in the HEI-OC1 group, the levels of PINK1, Parkin, P62, and LC3B were elevated in the HEI-OC1-AGEs and HEI-OC1-LacZ-AGEs groups. Compared with those in the HEI-OC1-LacZ group, the levels of PINK1, Parkin, P62, and LC3B in the HEI-OC1-LacZ-AGEs group also increased. Conversely, compared with those in HEI-OC1-LacZ-AGEs, the expression of PINK1, P62, and LC3B in HEI-OC1-Trx1-AGEs was lower (Fig. 5I, J).

### Inhibition of TOMM22 by CCCP reversed the protective effect of Trx1

**Fig. 6:**
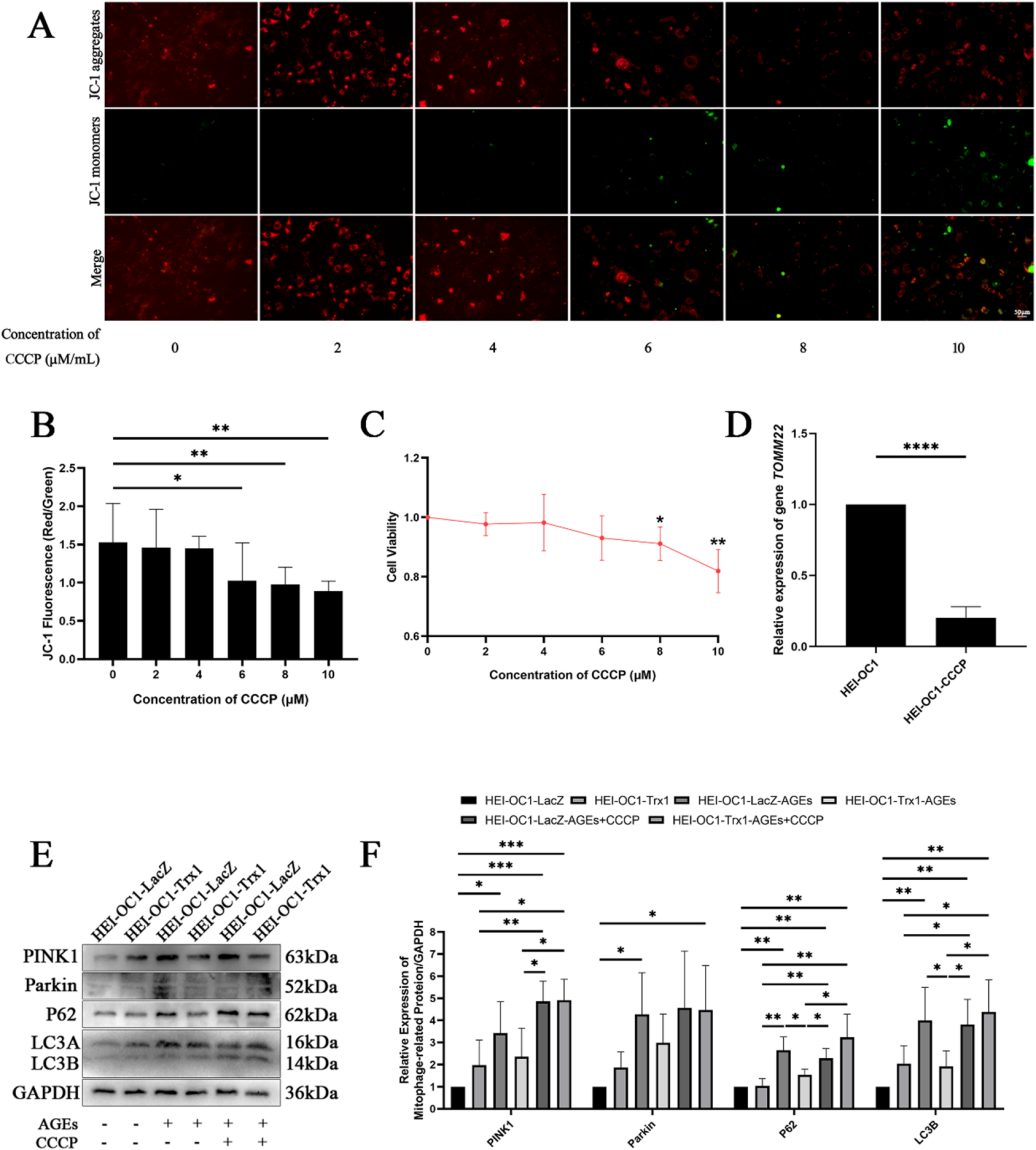
Inhibition of TOMM22 by CCCP reversed the protective effect of Trx1. (**A**,**B**) Changes in the mitochondrial membrane potential of HEI-OC1 cells after treatment with different concentrations of CCCP as determined by a JC-1 assay (Scale bar=50 μm. Images were taken at ×20 magnification). (**C**) The optimal effective concentration of CCCP as determined by a CCK-8 assay. (**D**) Expression of *TOMM22* in HEI-OC1 cells treated with CCCP as determined by RT‒qPCR. (**E**,**F**) Expression of mitochondrial autophagy-related proteins in HEI-OC1-Trx1/LacZ cells treated with CCCP and AGEs as determined by Western blotting. Western blotting and image presentation: Cropped blot images are displayed for clarity and conciseness. Within each horizontal panel, the lanes are contiguous on the original blot, representing samples with proteins of similar molecular weight analysed on the same gel. Vertically arranged panels group cropped sections from the same original membrane that were probed for different target proteins with distinct molecular weights. These vertical groups are explicitly separated by black horizontal dividing lines. Full, uncropped scans of all original blots, with groups clearly labelled, are provided in the Supplementary Information. *P < 0.05, **P < 0.01, ***P < 0.001, **** *P* < 0.0001.

The expression of *TOMM22* was inhibited by reducing the mitochondrial membrane potential via CCCP, and the optimal concentration was determined through JC-1 and CCK-8 assays. After 20 min of CCCP treatment, the results of the JC-1 assay indicated that compared with those in the 0 µM group, the red/green fluorescence ratio in the HEI-OC1 cells treated with 6, 8, and 10 µM CCCP significantly decreased (Fig. 6A, B). The results of the CCK-8 assay demonstrated that cell viability significantly decreased in HEI-OC1 cells treated with 8 and 10 µM CCCP (Fig. 6C). Consequently, 6 µM CCCP was selected as the optimal concentration, as it reduced the mitochondrial membrane potential in HEI-OC1 cells without causing noticeable cell damage. RT‒qPCR revealed a significant decrease in *TOMM22* expression in HEI-OC1 cells following CCCP treatment (Fig. 6D). Western blotting was conducted to detect the expression of mitochondrial autophagy-related proteins. Compared with those in the HEI-OC1-LacZ group, the expression levels of PINK1, Parkin, P62, and LC3B in the HEI-OC1-LacZ-AGEs group were greater. Conversely, compared with those in the HEI-OC1-LacZ-AGEs group, the expression of PINK1, P62, and LC3B in the HEI-OC1-Trx1-AGEs group were lower. Furthermore, the expression of PINK1, P62, and LC3B in the HEI-OC1-Trx1-AGEs-CCCP group was greater than that in the HEI-OC1-Trx1-AGEs group (Fig. 6E, F).

### Mechanism through which Trx1 overexpression regulates BV2 polarization to protect cochlear hair cells

**Fig. 7:**
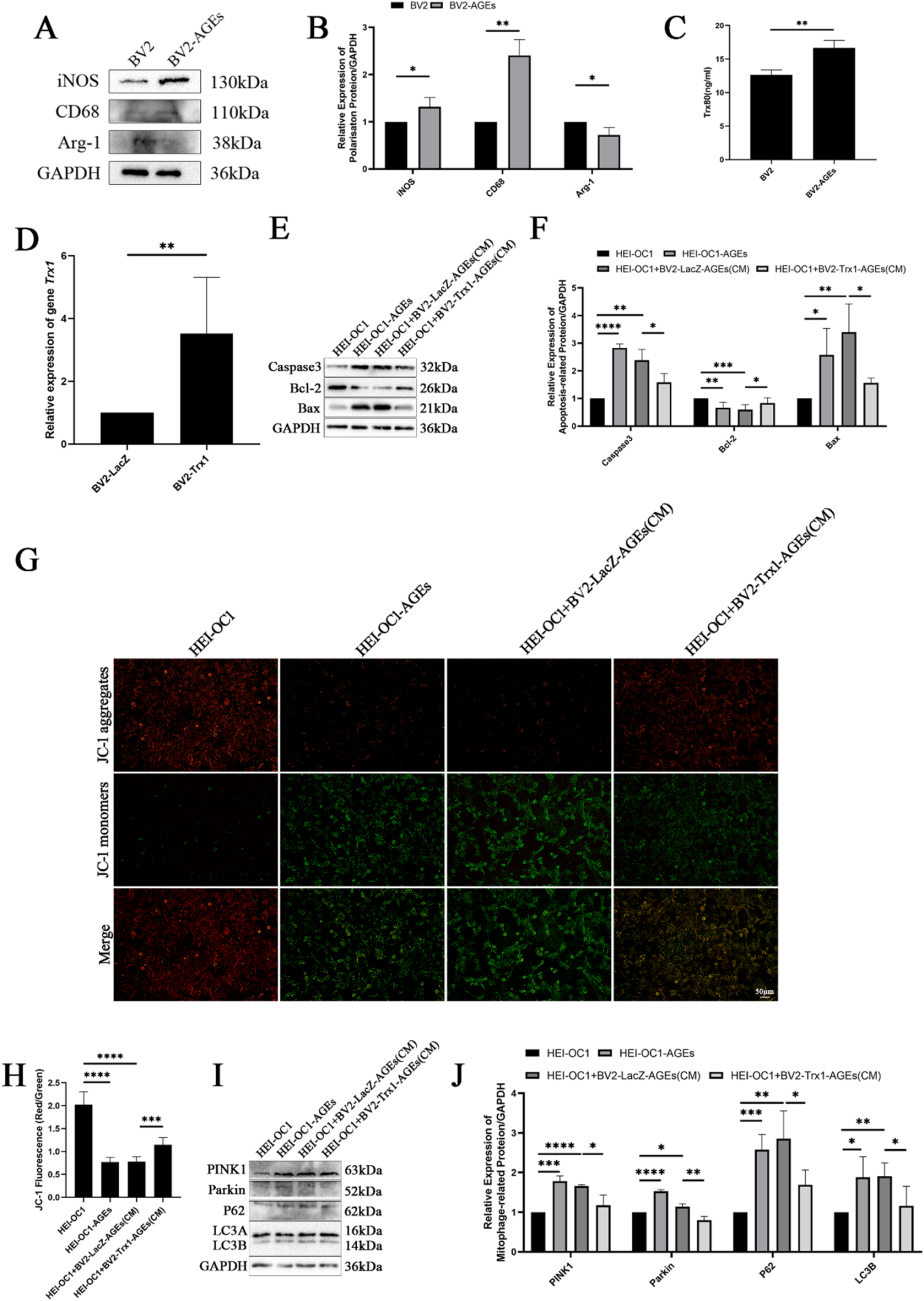
Mechanism through which Trx1 overexpression regulates BV2 polarization to protect cochlear hair cells. (**A**,**B**) Expression of polarization marker proteins in BV2 cells induced by AGEs determined by Western blotting. (**C**–**H**) Trx80 expression in BV2 cells treated with AGEs determined by ELISA. (**D**–**J**) Expression of *Trx1* in BV2 cells after transfection determined by RT‒qPCR. (**E**,**F**) Expression of apoptotic proteins in HEI-OC1 cells treated with BV2-Trx1/LacZ conditioned medium and AGEs determined by Western blotting. (**G**,**H**) Changes in the mitochondrial membrane potential in HEI-OC1 cells treated with BV2-Trx1/LacZ conditioned medium and AGEs determined by a JC-1 assay (Scale bar=50 μm. Images were taken at ×20 magnification). (**I**,**J**) Expression of mitochondrial autophagy-related proteins in HEI-OC1 cells treated with BV2-Trx1/LacZ conditioned medium and AGEs determined by Western blotting. Western blotting and image presentation: Cropped blot images are displayed for clarity and conciseness. Within each horizontal panel, the lanes are contiguous on the original blot, representing samples with proteins of similar molecular weight analysed on the same gel. Vertically arranged panels group cropped sections from the same original membrane that were probed for different target proteins with distinct molecular weights. These vertical groups are explicitly separated by black horizontal dividing lines. Full, uncropped scans of all original blots, with groups clearly labelled, are provided in the Supplementary Information. *P < 0.05, **P < 0.01, ***P < 0.001, **** *P* < 0.0001.

BV2 cell polarization was induced using AGEs. Western blotting revealed that in the BV2-AGEs group, the levels of the M1 polarization marker proteins inducible nitric oxide synthase (iNOS) and CD68 were elevated, whereas the expression of the M2 polarization marker protein arginase-1 (Arg-1) was reduced (Fig. 7A, B). ELISAs revealed an increase in the expression of Trx80 in the BV2-AGEs group (Fig. 7C). Successful transfection of BV2-Trx1 cells was confirmed by RT‒qPCR, which revealed increased expression of *Trx1* in BV2-Trx1 cells (Fig. 7D). AGEs were used to induce the polarization of BV2-LacZ and BV2-Trx1 cells, and the conditioned medium from these cultures, or high-glucose medium, was cocultured with HEI-OC1 cells at a ratio of 7:3. The expression of apoptosis-related proteins in HEI-OC1 cells was subsequently assessed via Western blotting. The results indicated that compared with those in the control group, the levels of the proapoptotic proteins Caspase3 and Bax in the HEI-OC1-AGEs and HEI-OC1-BV2-LacZ-AGEs (CM) groups were elevated, whereas the expression of the antiapoptotic protein Bcl-2 was reduced. Compared with those in the HEI-OC1-BV2-LacZ-AGEs (CM) group, the expression levels of the proapoptotic proteins Caspase3 and Bax in the HEI-OC1 cells in the HEI-OC1-BV2-Trx1-AGEs (CM) group were lower, while the expression of the antiapoptotic protein Bcl-2 was greater (Fig. 7E, F). The results of the JC-1 assay revealed a decrease in the ratio of red to green fluorescence in the HEI-OC1-AGEs and HEI-OC1-BV2-LacZ-AGEs (CM) groups compared with that in the HEI-OC1 group. Conversely, the ratio of red to green fluorescence in the HEI-OC1-BV2-Trx1-AGEs (CM) group increased compared with that in the HEI-OC1-BV2-LacZ-AGEs (CM) group (Fig. 7G, H). Western blotting was conducted to assess the expression of mitochondrial autophagy-related proteins in HEI-OC1 cells. Compared with those in the control group, the levels of the mitochondrial autophagy pathway proteins PINK1, Parkin, P62, and LC3B in the HEI-OC1-AGEs and HEI-OC1-BV2-LacZ-AGEs (CM) groups were greater. In contrast, the expression levels of PINK1, Parkin, P62, and LC3B in the HEI-OC1-BV2-Trx1-AGEs (CM) group were lower than those in the HEI-OC1-BV2-LacZ-AGEs (CM) group (Fig. 7I, J).

## Discussion

Oxidative stress resulting from the excessive generation of reactive oxygen species and free radicals in cells in high-glucose environments is a critical mechanism through which diabetes complications arise. The early prediction of tissue damage associated with diabetes, which involves the use of antioxidation-related indicators and the protection of corresponding target organs from an antioxidative perspective, has emerged as a novel approach for the diagnosis and treatment of diabetic complications^42–45^. This study revealed that patients in the DMHL group exhibited reductions in TEOAE and DPOAE, which suggests direct damage to hair cells in the context of diabetes. Serum Trx levels in individuals with type 2 diabetes were significantly elevated and negatively correlated with TEOAE and DPOAE levels, indicating that Trx may function as a sensitive serum marker for diabetic hearing loss. Importantly, the correlation between Trx and TEOAE parameters was stronger, suggesting that Trx may be more sensitive for the early assessment of hearing function. Our research revealed that elevated serum Trx levels are associated with a decline in cochlear function, which is consistent with the literature suggesting that elevated serum Trx levels are correlated with poor prognosis in patients with various diseases^46–50^. This phenomenon can be analysed from several perspectives. First, after Trx acts as an antioxidant within cells, it becomes inactive and is subsequently released into the bloodstream by tissues. Second, the oxidative stress response and antioxidant processes are interrelated, as the antioxidant effect is a response to oxidative stress within the body. An increase in antioxidant protein expression signifies an increase in the body’s response to oxidative stress, establishing a positive correlation between Trx levels and the degree of the oxidative stress response. Finally, elevated serum Trx levels indicate an uncontrolled response to oxidative stress. Excessive inhibition or enhancement of the antioxidant response fails to mitigate oxidative stress effectively. Consequently, the overexpression of Trx, an antioxidant protein, serves as a marker for disease progression and adverse outcomes. These findings directly associate Trx with the oxidative stress mechanisms underlying diabetic complications, thereby offering a potential target for noninvasive screening of diabetic hearing loss.

Under physiological conditions, PINK1 is imported into healthy mitochondria via a complex formed by the translocase of the outer mitochondrial membrane (TOMM) and the translocase of the inner mitochondrial membrane (TIMM). PINK1 is subsequently processed by PMPC and presenilin-associated rhomboid-like protease (PARL), which is associated with progerin. The loss of mitochondrial channel function, whether due to the collapse of the mitochondrial transmembrane potential (ΔΨ) or other factors, results in the accumulation of PINK1 at the outer mitochondrial membrane. This accumulation leads to the recruitment of Parkin to the vicinity of the TOMM complex, thereby initiating the mitochondrial autophagy pathway. The TOMM complex consists of seven subunits, with TOMM22 serving as the central component. TOMM22 is responsible for recognizing and transporting mitochondrial protein precursors synthesized in the cytoplasm and works in concert with TOMM20 to facilitate the transport of peptide receptors^51^. Through bioinformatics analysis, we determined that the PINK1-mediated mitochondrial autophagy pathway is highly conserved in both humans and mice. Analysis of the diabetic retinopathy dataset involved the construction of an interaction network among differentially expressed genes related to mitochondrial antioxidant stress and the PINK1 pathway. This analysis revealed that TOMM22 occupies a critical node within the interaction network involving Trx and the PINK1/Parkin/LC3B axis. These findings led to the generation of a novel scientific hypothesis: in individuals with diabetes, Trx may influence mitochondrial autophagy by regulating TOMM22. We subsequently verified the pathological changes in the diabetic cochlea and the regulatory role of Trx in animal models. In the diabetic mouse model, we observed apoptosis in cochlear tissue, as indicated by the upregulation of Caspase3 and Bax expression and the downregulation of Bcl-2 expression. This was accompanied by full activation of the mitochondrial autophagy pathway, as evidenced by the expression of PINK1, Parkin, P62, and LC3B and a decrease in *TOMM22* expression. However, in Trx-overexpressing mice (TgDM), these abnormal changes were significantly alleviated. This preliminary evidence suggests that the upregulation of Trx expression can inhibit cochlear apoptosis, neuroinflammation, and excessive mitochondrial autophagy induced by diabetes.

To elucidate the direct cellular autonomous mechanism underlying the protective effect of Trx, we examined it in the auditory hair cell model HEI-OC1. In individuals with type 2 diabetes mellitus (T2DM), persistently elevated glucose levels lead to an increase in advanced glycation end products (AGEs), a diverse group of compounds formed through the nonenzymatic glycosylation of proteins, lipids, and nucleic acids via the Maillard reaction^52^. In cellular experiments, we demonstrated that AGE-induced oxidative stress effectively mimicked the detrimental effects of the diabetic microenvironment on HEI-OC1 cells. This damage was evidenced by a reduction in the mitochondrial membrane potential, activation of apoptotic proteins, abnormal enhancement of mitochondrial autophagy, and downregulation of *TOMM22* expression. These results align with the literature that describes the disruption of the mitochondrial quality control system in the context of type 2 diabetes^53–55^. *Trx1* overexpression markedly reversed these alterations, restored the mitochondrial membrane potential, decreased apoptosis, suppressed the excessive activation of the PINK1/Parkin pathway, and increased the expression level of *TOMM22*. These findings establish a clear connection between *TOMM22* expression and the activation of mitochondrial autophagy as well as cell fate. Studies have demonstrated that TOMM22 serves as a novel regulatory factor for mitochondrial dynamics and bioenergetic function, which are essential for restoring mitochondrial dynamics and ensuring the survival of vascular endothelial cells upon exposure to high concentrations of glucose^56^. To further investigate the role of TOMM22 in the Trx-mediated protective pathway, we conducted a CCCP intervention experiment designed to specifically reduce the mitochondrial membrane potential and inhibit *TOMM22* expression. The results indicated that mitochondrial autophagy markers (PINK1, P62, and LC3B), whose expression was initially suppressed because of *Trx* overexpression, were reactivated. These findings confirm that Trx inhibits excessive mitochondrial autophagy by maintaining *TOMM22* expression, highlighting its critical role in this protective mechanism.

Inner ear microglia-like cells were first identified in the inner ears of birds and mice. In 2008, Okano et al. demonstrated the presence of bone marrow-derived cells expressing the microglia-specific marker Iba-1 in the inner ear of mice, where they manifested as tissue-resident macrophages^57^. O’Malley et al. subsequently reported the presence of Iba-1+, CD68+, and CD163 + macrophages/microglia in the adult cochlea^58^. Seigel et al. isolated and enriched CD11b+ cell populations from the cochlea, ultimately immortalizing these cells to establish a novel microglial cell line known as Mocha (cochlear microglia)^59^. Dendritic microglia-like cells were observed among the damaged auditory sensory epithelial cells in the pre-OHC region following neomycin injury. These findings suggest that microglia-like cells are involved in the processing of degenerative cell debris and the direct repair of damaged auditory sensory epithelium^26^. Furthermore, microglial inflammatory responses increase with the progression of age-related hearing loss (ARHL) and are correlated with the severity of hearing impairment^60^. Our research revealed a novel pathway through which Trx indirectly safeguards auditory cells by modulating the neuroimmune microenvironment, as demonstrated by coculture experiments with conditioned medium. We observed that advanced glycation end products (AGEs) induced BV2 cells to polarize towards the M1 proinflammatory phenotype, as indicated by the upregulation of M1 marker proteins such as iNOS and CD68. Furthermore, the overexpression of Trx facilitated the transition of these cells to the M2 anti-inflammatory phenotype, characterized by the upregulation of the M2 marker protein Arg-1. These findings are consistent with the results of Wang AL et al., who reported that AGEs can activate rat retinal microglia and promote the expression and release of TNF-α^61^. Notably, we observed a significant increase in Trx80 expression in BV2 cells under AGE stimulation, providing direct evidence for the association between Trx system imbalance and microglial activation. We also found that in response to AGE stimulation, hair cells produce Trx80 on their own, which is consistent with the findings of Fujioka et al., who reported that nonmacrophage- or microglia-mediated inflammatory responses and the release of proinflammatory cytokines in the early stages of damage, such as excessive noise stimulation, occur in the inner ear^62^. These findings suggest that in the pathological microenvironment, damaged hair cells may actively participate in and amplify local inflammatory responses through the secretion of proinflammatory signalling molecules such as Trx80, resulting in the formation of an “injury–inflammation–reinjury” positive feedback loop. The conditioned medium of BV2 cells with high Trx1 expression can significantly alleviate mitochondrial dysfunction and excessive autophagy in HEI-OC1 cells, indicating that Trx not only directly protects hair cells but also creates an anti-inflammatory microenvironment conducive to hair cell survival through the regulation of adjacent microglia.

This research has yielded innovative discoveries; however, certain limitations persist. While this study revealed a correlation between serum Trx levels and hearing function, this correlation remains a cross-sectional association. The potential of Trx as a longitudinal biomarker for predicting the onset or progression of diabetic hearing loss requires validation through larger scale prospective cohort studies. Furthermore, this study revealed associations among the Trx1, TOMM22, and PINK1/Parkin pathways; however, the precise mechanism through which Trx regulates the molecular bridge of TOMM22 is not fully understood. Trx may stabilize the TOMM22 protein through direct interactions or modulate its expression by influencing transcription factors, both of which warrant further investigation. Recent studies have indicated that redox-sensitive proteins, such as DJ-1, play a critical role in cellular protection by regulating the stability of mitochondrial membrane proteins^63,64^. This finding offers a promising avenue for investigating the mechanism by which Trx regulates TOMM22. Additionally, our research demonstrated that inhibiting excessive mitochondrial autophagy has a protective effect. However, a significant challenge in translational medicine lies in the precise regulation of this activity under pathological conditions without disruption of normal cellular functions.

In summary, this study systematically elucidates the protective role of thioredoxin in diabetic hearing loss and its multifaceted mechanisms through clinical observations, bioinformatics analyses, animal models, and multilevel cellular experiments. Thioredoxin maintains mitochondrial homeostasis by directly regulating the TOMM22-PINK1/Parkin axis and improves the auditory microenvironment by modulating microglial polarization. These findings not only advance the understanding of the pathogenesis of diabetic hearing loss but also provide a theoretical foundation for developing intervention strategies targeting the Trx pathway. Future research may further investigate the translational potential of Trx and its derivatives in preventing and treating diabetic hearing complications.

## Conclusion

**Fig. 8:**
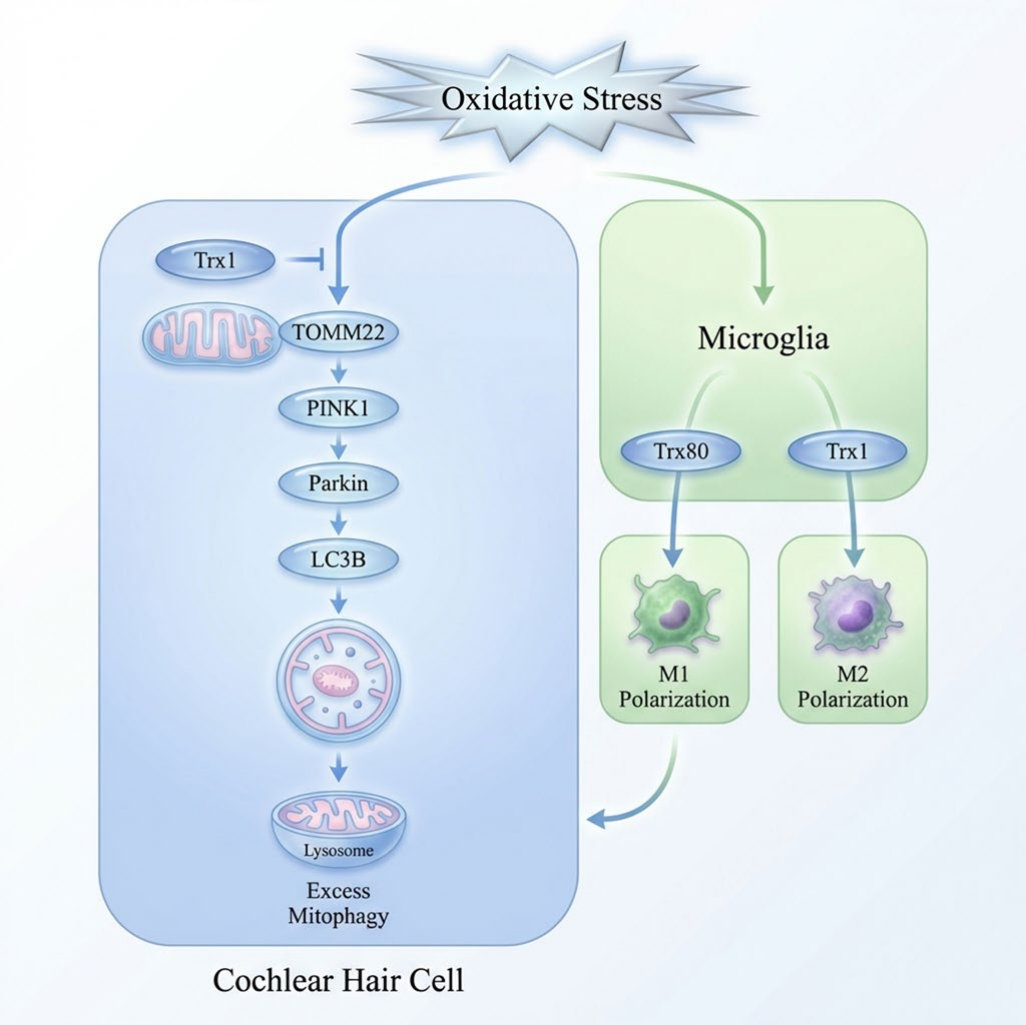
Comprehensive overview of the protective effect of Trx1 on diabetes-related hearing loss through the regulation of mitophagy and microglial polarization.

Serum Trx levels represent a potentially sensitive biomarker for diabetic hearing impairment. Trx plays a crucial role in maintaining mitochondrial homeostasis and preventing excessive mitochondrial autophagy and apoptosis in auditory hair cells by regulating the expression of the mitochondrial outer membrane protein TOMM22 and inhibiting the overactivation of the PINK1/Parkin pathway in the context of diabetes. Furthermore, Trx modulates microglial polarization, facilitating the transition from the proinflammatory M1 phenotype to the anti-inflammatory M2 phenotype. The resulting beneficial microenvironment indirectly safeguards auditory hair cells from diabetes-related damage (Fig. 8).

## Supplementary Information

Below is the link to the electronic supplementary material.

Supplementary Material 1

## Data Availability

The datasets generated during and/or analysed during the current study are available from the corresponding author on reasonable request.
