# Supplementary material for: Thioredoxin protects against diabetic hearing loss by regulating TOMM22 mediated mitochondrial autophagy in hair cells and inhibiting microglial M1 polarization
Source: Sci Rep. 2026 Mar 20;16:14332. doi: 10.1038/s41598-026-44909-3 (PMC13144412; doi:10.1038/s41598-026-44909-3)
Supplement: Supplementary file 1 — Supplementary Material 1 [file 41598_2026_44909_MOESM1_ESM.pdf]

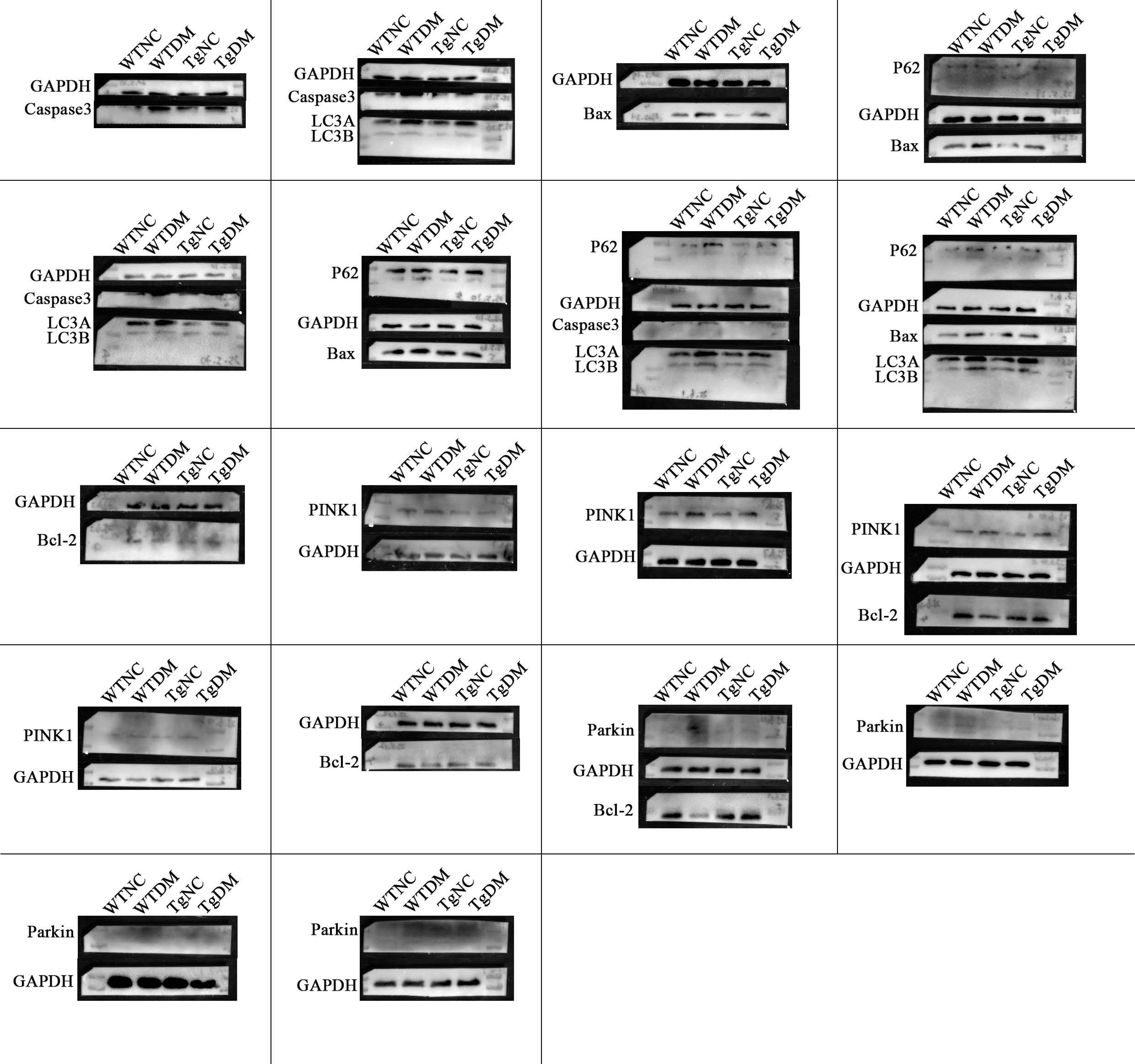
WTNC WTDM TgNC TgDM
GAPDH
Caspase3
WTNC WTDM TgNC TgDM
GAPDH
Caspase3
LC3A
LC3B
WTNC WTDM TgNC TgDM
GAPDH
Bax
WTNC WTDM TgNC TgDM
P62
GAPDH
Bax
WTNC WTDM TgNC TgDM
GAPDH
Caspase3
LC3A
LC3B
WTNC WTDM TgNC TgDM
P62
GAPDH
Bax
WTNC WTDM TgNC TgDM
P62
GAPDH
Caspase3
LC3A
LC3B
WTNC WTDM TgNC TgDM
P62
GAPDH
Bax
LC3A
LC3B
WTNC WTDM TgNC TgDM
GAPDH
Bcl-2
WTNC WTDM TgNC TgDM
PINK1
GAPDH
WTNC WTDM TgNC TgDM
PINK1
GAPDH
WTNC WTDM TgNC TgDM
PINK1
GAPDH
Bcl-2
WTNC WTDM TgNC TgDM
PINK1
GAPDH
WTNC WTDM TgNC TgDM
GAPDH
Bcl-2
WTNC WTDM TgNC TgDM
Parkin
GAPDH
Bcl-2
WTNC WTDM TgNC TgDM
Parkin
GAPDH
WTNC WTDM TgNC TgDM
Parkin
GAPDH
WTNC WTDM TgNC TgDM
Parkin
GAPDH

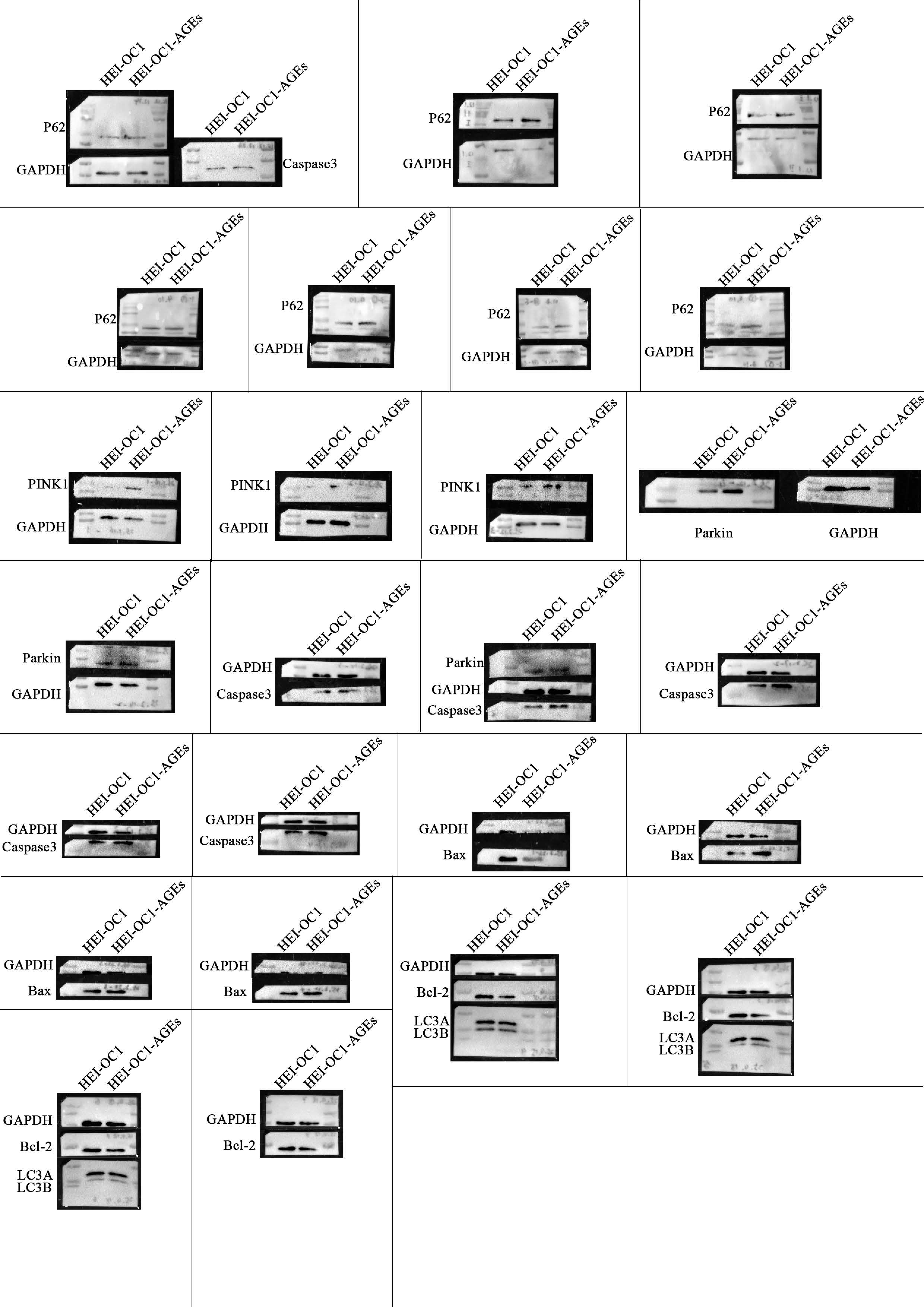
HEI-OC1
HEI-OC1-AGEs
P62
GAPDH
HEI-OC1
HEI-OC1-AGEs
Caspase3
HEI-OC1
HEI-OC1-AGEs
P62
GAPDH
HEI-OC1
HEI-OC1-AGEs
P62
GAPDH
HEI-OC1
HEI-OC1-AGEs
P62
GAPDH
HEI-OC1
HEI-OC1-AGEs
P62
GAPDH
HEI-OC1
HEI-OC1-AGEs
P62
GAPDH
HEI-OC1
HEI-OC1-AGEs
P62
GAPDH
HEI-OC1
HEI-OC1-AGEs
PINK1
GAPDH
HEI-OC1
HEI-OC1-AGEs
PINK1
GAPDH
HEI-OC1
HEI-OC1-AGEs
PINK1
GAPDH
HEI-OC1
HEI-OC1-AGEs
HEI-OC1
HEI-OC1-AGEs
Parkin
GAPDH
HEI-OC1
HEI-OC1-AGEs
Parkin
GAPDH
HEI-OC1
HEI-OC1-AGEs
GAPDH
Caspase3
HEI-OC1
HEI-OC1-AGEs
Parkin
GAPDH
Caspase3
HEI-OC1
HEI-OC1-AGEs
GAPDH
Caspase3
HEI-OC1
HEI-OC1-AGEs
GAPDH
Caspase3
HEI-OC1
HEI-OC1-AGEs
GAPDH
Caspase3
HEI-OC1
HEI-OC1-AGEs
GAPDH
Bax
HEI-OC1
HEI-OC1-AGEs
GAPDH
Bax
HEI-OC1
HEI-OC1-AGEs
GAPDH
Bax
HEI-OC1
HEI-OC1-AGEs
GAPDH
Bax
HEI-OC1
HEI-OC1-AGEs
GAPDH
Bcl-2
LC3A
LC3B
HEI-OC1
HEI-OC1-AGEs
GAPDH
Bcl-2
LC3A
LC3B
HEI-OC1
HEI-OC1-AGEs
GAPDH
Bcl-2
LC3A
LC3B
HEI-OC1
HEI-OC1-AGEs
GAPDH
Bcl-2

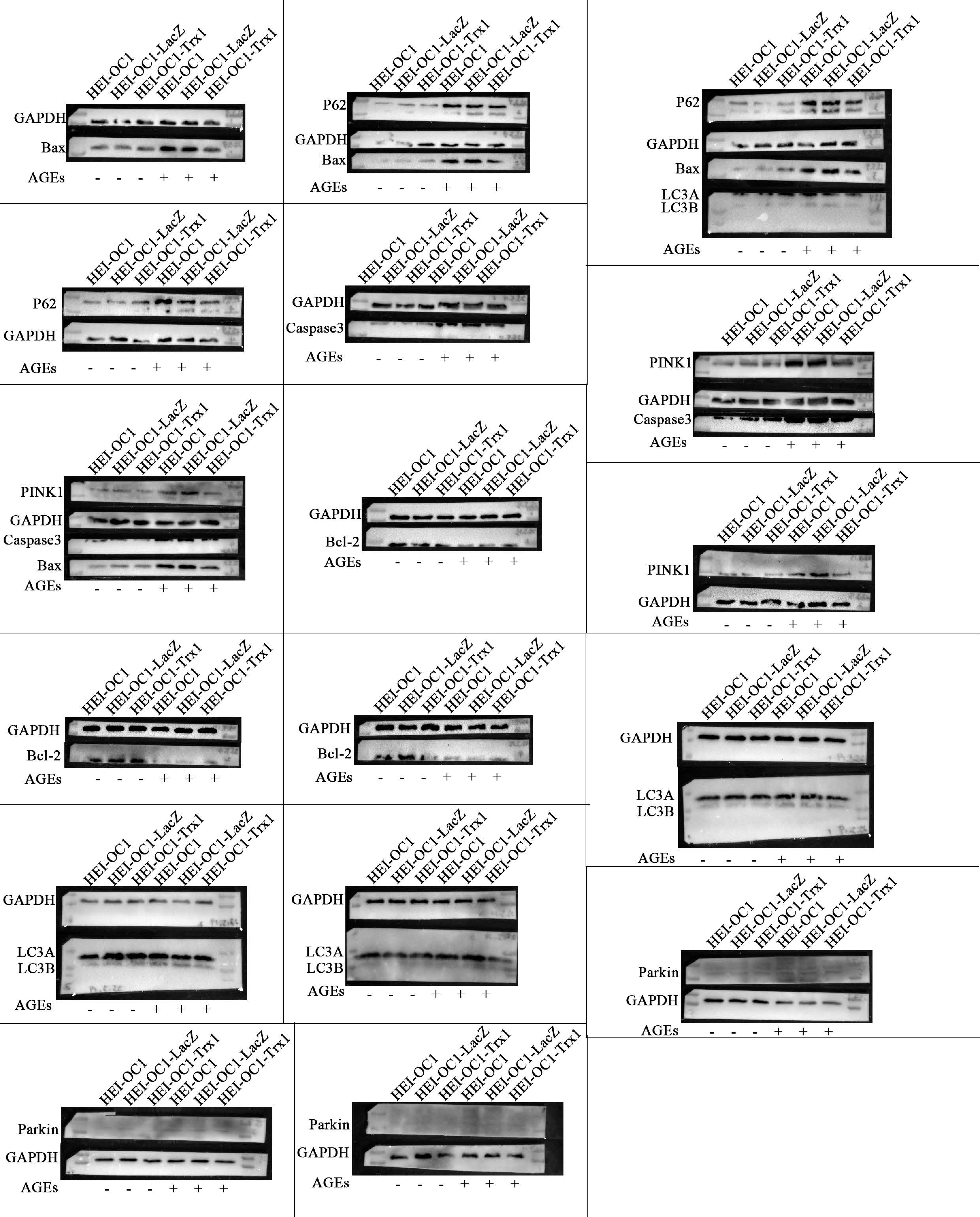
HEI-OC1
HEI-OC1-LacZ
HEI-OC1-Trx1
HEI-OC1
HEI-OC1-LacZ
HEI-OC1-Trx1
GAPDH
Bax
AGEs - - - + + +
HEI-OC1
HEI-OC1-LacZ
HEI-OC1-Trx1
HEI-OC1
HEI-OC1-LacZ
HEI-OC1-Trx1
P62
GAPDH
Bax
AGEs - - - + + +
HEI-OC1
HEI-OC1-LacZ
HEI-OC1-Trx1
HEI-OC1
HEI-OC1-LacZ
HEI-OC1-Trx1
P62
GAPDH
Bax
LC3A
LC3B
AGEs - - - + + +
HEI-OC1
HEI-OC1-LacZ
HEI-OC1-Trx1
HEI-OC1
HEI-OC1-LacZ
HEI-OC1-Trx1
P62
GAPDH
AGEs - - - + + +
HEI-OC1
HEI-OC1-LacZ
HEI-OC1-Trx1
HEI-OC1
HEI-OC1-LacZ
HEI-OC1-Trx1
GAPDH
Caspase3
AGEs - - - + + +
HEI-OC1
HEI-OC1-LacZ
HEI-OC1-Trx1
HEI-OC1
HEI-OC1-LacZ
HEI-OC1-Trx1
PINK1
GAPDH
Caspase3
AGEs - - - + + +
HEI-OC1
HEI-OC1-LacZ
HEI-OC1-Trx1
HEI-OC1
HEI-OC1-LacZ
HEI-OC1-Trx1
PINK1
GAPDH
Caspase3
Bax
AGEs - - - + + +
HEI-OC1
HEI-OC1-LacZ
HEI-OC1-Trx1
HEI-OC1
HEI-OC1-LacZ
HEI-OC1-Trx1
GAPDH
Bcl-2
AGEs - - - + + +
HEI-OC1
HEI-OC1-LacZ
HEI-OC1-Trx1
HEI-OC1
HEI-OC1-LacZ
HEI-OC1-Trx1
PINK1
GAPDH
AGEs - - - + + +
HEI-OC1
HEI-OC1-LacZ
HEI-OC1-Trx1
HEI-OC1
HEI-OC1-LacZ
HEI-OC1-Trx1
GAPDH
Bcl-2
AGEs - - - + + +
HEI-OC1
HEI-OC1-LacZ
HEI-OC1-Trx1
HEI-OC1
HEI-OC1-LacZ
HEI-OC1-Trx1
GAPDH
Bcl-2
AGEs - - - + + +
HEI-OC1
HEI-OC1-LacZ
HEI-OC1-Trx1
HEI-OC1
HEI-OC1-LacZ
HEI-OC1-Trx1
GAPDH
LC3A
LC3B
AGEs - - - + + +
HEI-OC1
HEI-OC1-LacZ
HEI-OC1-Trx1
HEI-OC1
HEI-OC1-LacZ
HEI-OC1-Trx1
GAPDH
LC3A
LC3B
AGEs - - - + + +
HEI-OC1
HEI-OC1-LacZ
HEI-OC1-Trx1
HEI-OC1
HEI-OC1-LacZ
HEI-OC1-Trx1
GAPDH
LC3A
LC3B
AGEs - - - + + +
HEI-OC1
HEI-OC1-LacZ
HEI-OC1-Trx1
HEI-OC1
HEI-OC1-LacZ
HEI-OC1-Trx1
Parkin
GAPDH
AGEs - - - + + +
HEI-OC1
HEI-OC1-LacZ
HEI-OC1-Trx1
HEI-OC1
HEI-OC1-LacZ
HEI-OC1-Trx1
Parkin
GAPDH
AGEs - - - + + +
HEI-OC1
HEI-OC1-LacZ
HEI-OC1-Trx1
HEI-OC1
HEI-OC1-LacZ
HEI-OC1-Trx1
Parkin
GAPDH
AGEs - - - + + +

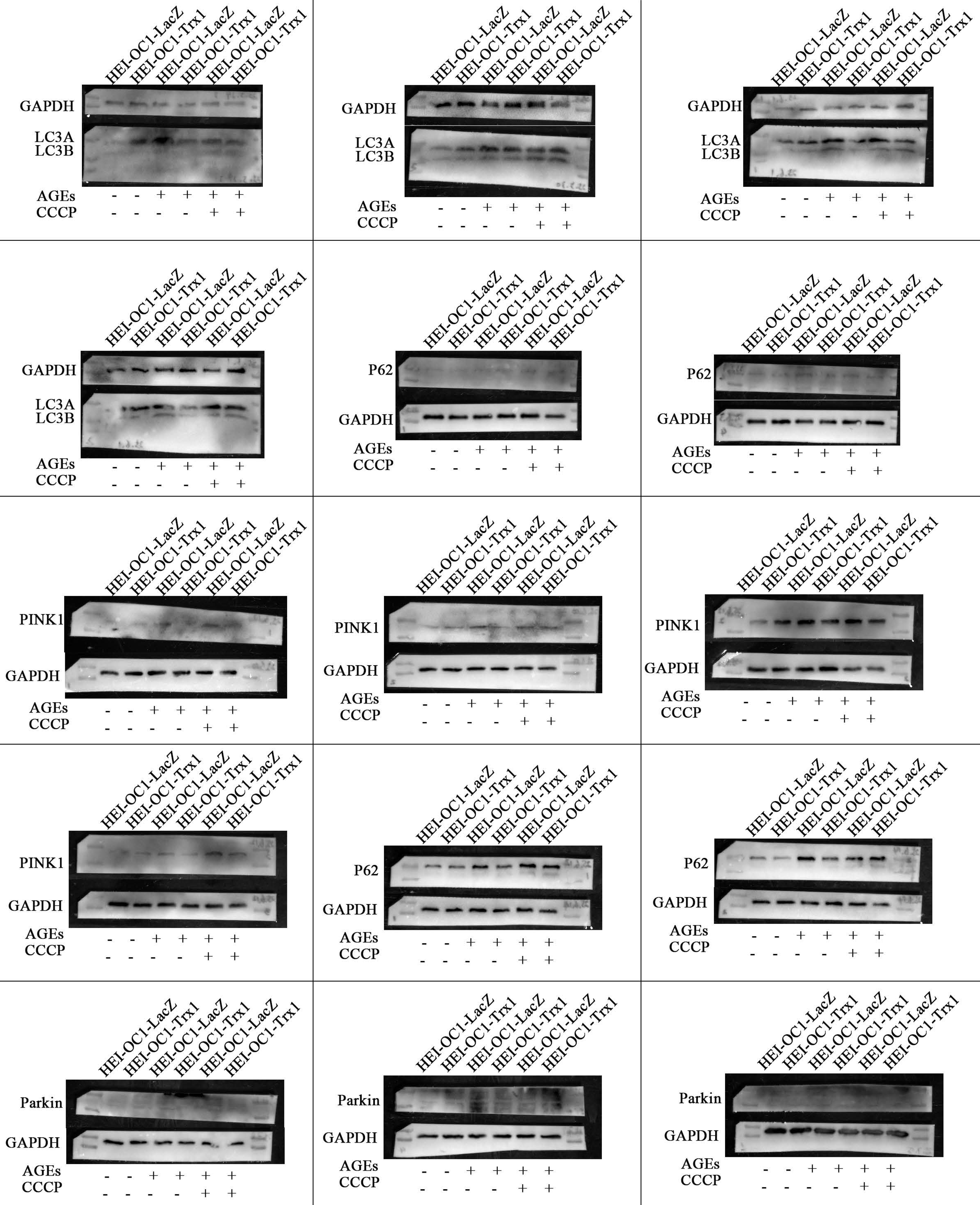

HEI-OC1-LacZ HEI-OC1-Trx1 HEI-OC1-LacZ HEI-OC1-Trx1 HEI-OC1-LacZ HEI-OC1-Trx1
GAPDH
LC3A
LC3B
AGEs - - + + + +
CCCP - - - - + +
HEI-OC1-LacZ HEI-OC1-Trx1 HEI-OC1-LacZ HEI-OC1-Trx1 HEI-OC1-LacZ HEI-OC1-Trx1
GAPDH
LC3A
LC3B
AGEs - - + + + +
CCCP - - - - + +
HEI-OC1-LacZ HEI-OC1-Trx1 HEI-OC1-LacZ HEI-OC1-Trx1 HEI-OC1-LacZ HEI-OC1-Trx1
GAPDH
LC3A
LC3B
AGEs - - + + + +
CCCP - - - - + +
HEI-OC1-LacZ HEI-OC1-Trx1 HEI-OC1-LacZ HEI-OC1-Trx1 HEI-OC1-LacZ HEI-OC1-Trx1
GAPDH
LC3A
LC3B
AGEs - - + + + +
CCCP - - - - + +
HEI-OC1-LacZ HEI-OC1-Trx1 HEI-OC1-LacZ HEI-OC1-Trx1 HEI-OC1-LacZ HEI-OC1-Trx1
P62
GAPDH
AGEs - - + + + +
CCCP - - - - + +
HEI-OC1-LacZ HEI-OC1-Trx1 HEI-OC1-LacZ HEI-OC1-Trx1 HEI-OC1-LacZ HEI-OC1-Trx1
P62
GAPDH
AGEs - - + + + +
CCCP - - - - + +
HEI-OC1-LacZ HEI-OC1-Trx1 HEI-OC1-LacZ HEI-OC1-Trx1 HEI-OC1-LacZ HEI-OC1-Trx1
PINK1
GAPDH
AGEs - - + + + +
CCCP - - - - + +
HEI-OC1-LacZ HEI-OC1-Trx1 HEI-OC1-LacZ HEI-OC1-Trx1 HEI-OC1-LacZ HEI-OC1-Trx1
PINK1
GAPDH
AGEs - - + + + +
CCCP - - - - + +
HEI-OC1-LacZ HEI-OC1-Trx1 HEI-OC1-LacZ HEI-OC1-Trx1 HEI-OC1-LacZ HEI-OC1-Trx1
PINK1
GAPDH
AGEs - - + + + +
CCCP - - - - + +
HEI-OC1-LacZ HEI-OC1-Trx1 HEI-OC1-LacZ HEI-OC1-Trx1 HEI-OC1-LacZ HEI-OC1-Trx1
PINK1
GAPDH
AGEs - - + + + +
CCCP - - - - + +
HEI-OC1-LacZ HEI-OC1-Trx1 HEI-OC1-LacZ HEI-OC1-Trx1 HEI-OC1-LacZ HEI-OC1-Trx1
P62
GAPDH
AGEs - - + + + +
CCCP - - - - + +
HEI-OC1-LacZ HEI-OC1-Trx1 HEI-OC1-LacZ HEI-OC1-Trx1 HEI-OC1-LacZ HEI-OC1-Trx1
P62
GAPDH
AGEs - - + + + +
CCCP - - - - + +
HEI-OC1-LacZ HEI-OC1-Trx1 HEI-OC1-LacZ HEI-OC1-Trx1 HEI-OC1-LacZ HEI-OC1-Trx1
Parkin
GAPDH
AGEs - - + + + +
CCCP - - - - + +
HEI-OC1-LacZ HEI-OC1-Trx1 HEI-OC1-LacZ HEI-OC1-Trx1 HEI-OC1-LacZ HEI-OC1-Trx1
Parkin
GAPDH
AGEs - - + + + +
CCCP - - - - + +
HEI-OC1-LacZ HEI-OC1-Trx1 HEI-OC1-LacZ HEI-OC1-Trx1 HEI-OC1-LacZ HEI-OC1-Trx1
Parkin
GAPDH
AGEs - - + + + +
CCCP - - - - + +

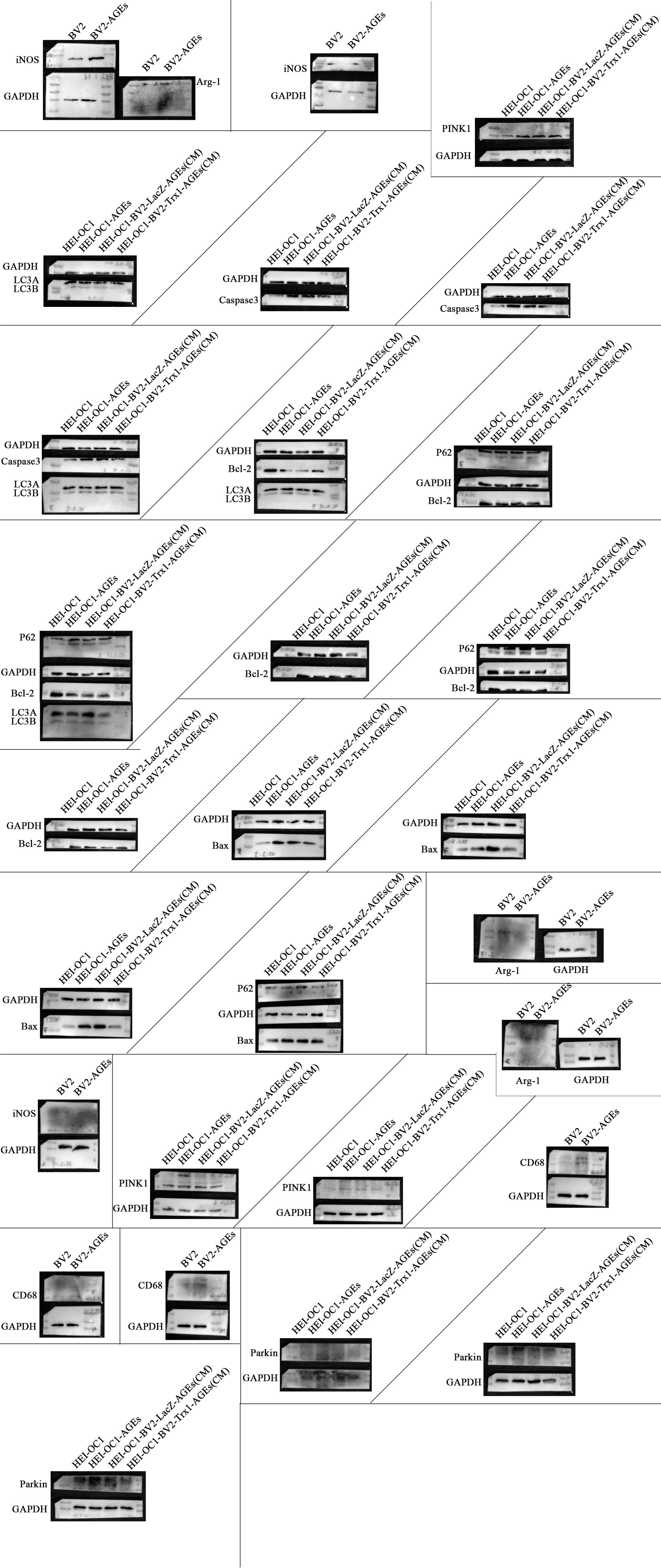

BV2
BV2-AGEs
iNOS
GAPDH
Arg-1
HEI-OC1
HEI-OC1-AGEs
HEI-OC1-BV2-LacZ-AGEs(CM)
HEI-OC1-BV2-Trx1-AGEs(CM)
PINK1
LC3A
LC3B
Caspase3
Bcl-2
P62
Bax
CD68
Parkin
